# Evaluating the impact of an oral nutrition supplement on biochemical profile, growth, and body composition in Indian children: an *in-silico* study

**DOI:** 10.3389/fnut.2025.1519756

**Published:** 2025-03-27

**Authors:** Sahana Sringari, Surabhi Vijaykumar, Manali Sherkane, Megha Venkatesh, Suranjana Gupta, Karan Lomore, K. V. Venkatesh

**Affiliations:** ^1^MetFlux Research Private Limited, Bengaluru, India; ^2^Department of Chemical Engineering, Institute of Technology Bombay, Mumbai, India

**Keywords:** biochemical profile, body composition, child growth, *in silico* study, oral nutrition supplement

## Abstract

**Background:**

*In-silico* modeling provides a valuable approach for predicting the effects of nutritional interventions on child growth, particularly in settings where large-scale human trials are challenging. Validated, population-level predictive platforms optimize dosing and intervention strategies, facilitating the development of targeted nutritional approaches that enhance growth outcomes in children.

**Objective:**

This study aims to assess the impact of an oral nutrition supplement (ONS) intervention on biochemical and growth parameters of Indian children using an *in-silico* intervention approach.

**Methods:**

An energetics-based modeling framework was developed to simulate the growth trajectories of children aged 6–16 years, integrating national datasets for Indian children. The model, validated with published literature, was designed to predict the effects of nutritional interventions. This study evaluated the impact of two Horlicks Oral Nutrition Supplement (ONS) formulations with varying micronutrient dosages on key growth outcomes. Various intervention scenarios were simulated, including comparisons of ONS with water vs. milk, and interventions with different nutrient compositions, such as macronutrients alone or a combination of macro- and micronutrients. The primary outcomes of the study focused on both biochemical and physical growth changes. Key serum nutrient levels were analyzed, alongside anthropometric measures such as height, weight, and body composition indicators, including fat-free mass, fat mass, and bone mineral content, over simulated periods of 4, 8, and 12 months.

**Results:**

The *in-silico* analysis predicted that two servings of Horlicks with milk significantly improved anthropometric and body composition parameters compared to both milk alone and other experimental groups. Biochemically, the Horlicks intervention led to notable increases in serum nutrient levels, which correlated with higher growth velocities and enhanced body composition relative to plain milk. The model underscored the critical role of combined macro- and micronutrient supplementation, with two servings yielding more pronounced effects than one.

**Conclusion:**

This study provides important insights into the potential benefits of Horlicks interventions for enhancing child growth outcomes. It underscores the effectiveness of computational models in the preliminary assessment of nutrition interventions, providing foundation for targeted clinical studies to improve child health and development. However, it is important to note that the model benchmarking was conducted using data from the Indian population, and the findings may not be directly applicable to other ethnicities without further validation.

## 1 Introduction

Children and adolescents undergo dynamic periods of development marked by substantial growth. This period is characterized by increment in height, bone mass, and musculature which necessitate a corresponding increase in energy and nutrient intake. Deficiencies in meeting these heightened nutrient requirements render children and adolescents vulnerable to nutritional deficiencies, potentially leading to varying degrees of malnutrition (1, 2). This age-group hence requires regular assessment and monitoring of nutritional status to ensure optimal growth and development. At the population level, such monitoring serves as a critical first step in benchmarking child growth and managing nutrition strategies. Additionally, it allows for the analysis of secular trends in child growth and nutrition, helping to identify patterns and areas in need of intervention. In Indian context, growth charts published by Indian Association of Pediatrics (IAP) (3) can help monitor normal, undernourished and overnourished growth trajectories, surveys like Comprehensive National Nutrition Survey (CNNS) (4) help assess nutritional status of children and adolescents. Availability of such resources become valuable in guiding interventions to address children and adolescents based on their specific nutritional status.

The 2018 CNNS reports that among Indian children aged 5–19 years, stunting affects 20–22%, wasting 23–24%, and overweight 4–5% (4). Additionally, secondary analysis of the CNNS data observes stunting prevalence is higher in late adolescence (30%) compared to early adolescence (25.6%), indicating an increasing burden of undernutrition as children transition into later developmental stages (5). The CNNS survey further reports notable prevalence of nutritional deficiencies. 19–25% of 5- to 19-year-old children were reported to be Vitamin A deficient, 28–38% were deficient in Vitamin B9, and 15–34% deficient in Vitamin B12. Furthermore, the prevalence percentages of iron, zinc, vitamin D and iodine deficiencies were 14–25%, 16–32%, 16–21%, and 4–6%, respectively (4). Another nationwide multi-center trial revealed that the prevalence of selenium and calcium deficiencies accounted for ~5 and 30%, respectively, in 6- to 16-year-olds (6).

Concerning prevalence of nutrient deficiencies and poor nutritional status among Indian children is attributed majorly to the lack of dietary diversity (7, 8). Several studies have employed milk as a dietary intervention to address childhood malnutrition and to promote dietary diversification given its rich nutrient profile (9–13). Milk is a significant source of high-quality protein and key micronutrients, essential for tissue growth and repair particularly during critical growth periods (14, 15). However, existing deficiencies can limit a child's ability to absorb these essential nutrients from unfortified milk. Enhancing nutrient profile of milk with multiple micronutrients or oral nutrition supplements (ONS) has potential to not only tackle micronutrient deficiencies but also to improve anthropometric indices in children and adolescents in children with inadequate dietary intake (16, 17). Previous studies on such interventions underline the importance of both; macro as well as micronutrients adequacy in improving growth trajectories of children and adolescents (18–22). However, evaluating these effects through large-scale clinical trials can be both resource-intensive and time-consuming. This challenge becomes even more pronounced in resource-limited settings, such as in India.

In this context, *in-silico* models present an efficient and practical alternative. These models simplify the assessment of growth processes by specifically focusing on key elements like nutrient intake and its direct influence on target outcomes. They also serve as a data analytics tool which can effectively deliver realistic growth predictions under defined conditions (23). The ability to simulate various scenarios allows preliminary assessment of interventions, ultimately offering actionable insights that can inform targeted nutritional strategies.

Given the limited studies on the effects of ONS interventions in milk on anthropometric and body composition parameters in Indian children, this study aims to bridge that gap using a system biology-based mathematical modeling and simulation approach. To achieve this, we utilized a predictive platform grounded in an integrated framework that draws on national datasets, along with data from published epidemiological studies and clinical trials. This comprehensive framework was designed to benchmark and validate child growth trajectory predictions in response to diverse nutritional interventions, offering a robust tool for assessing the efficacy of dietary strategies.

The study aims to evaluate the effects of two Horlicks compositions, with and without milk, on biochemical parameters and growth outcomes such as height velocity, weight velocity, BMI, fat-free mass (FFM), fat mass (FM), and bone mineral content (BMC) in children aged 6–16 years. This age range aligns with the CNNS survey population, allowing the simulation to reflect real-world nutritional gaps, while highlighting how targeted nutritional interventions can enhance physical development through *in-silico* modeling.

## 2 Materials and methods

### 2.1 Model framework and population simulation

**Figure 1:**
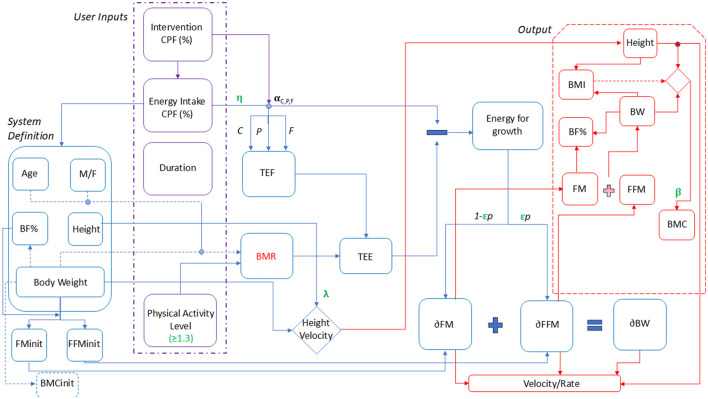
Child growth model representation. M/F, Male/Female; BF%, Body fat percentage; BW, Body weight; FM init, Initial fat mass; FFMinit, Initial fat-free mass; BMC init, Initial bone mineral content; CPF%, Carbohydrate, Protein, Fat percentage; RDA, Recommended Dietary Allowance; TEF, Thermic effect of Food; BMR, Basal Metabolic Rate.

To simulate a population of Indian children and adolescents, virtual individuals aged between 6 and 16 years were generated using an energetics-based phenomenological model. This model was benchmarked against the Indian Association of Pediatrics (IAP) 2015 growth data (3). The input variables for the model were defined as age, gender, height, and body weight, which together formed the system for simulating growth outcomes and responses to the intervention (Figure 1). The input body fat percentage (BF%) for different growth percentiles was taken from Khadilkar et al. which was further used to estimate FM and FFM (24). BMC was calculated based on published correlation (25).

To integrate nutrient intake to the above benchmarking of growth trajectory, the net energy intake levels was adjusted for body weight, as per the 2020 Recommended Dietary Allowances (RDA) for children and adolescents (26) and the energy balance was carried out on per day basis. Basal metabolic rate (BMR) was established while benchmarking to fit the growth percentile for each age and gender, with the above assumptions to obtain a similar growth percentile in the following year. The initial macronutrient composition was assumed to be 66% carbohydrates, 26% fat and 8% protein based on the observations from epidemiological studies (27–31). The physical activity was considered as per ICMR determined average physical activity levels (PAL) in the range of 1.4–1.67. Based on the net energy intake per day, the total energy expenditure was obtained considering macronutrient dependent thermic effect of food (TEF), PAL, and the BMR. The residual energy was used to calculate the increment in fat mass (δ FM) and fat free mass (δ FFM) defined by distribution coefficient “p” in turn the weight increment over a day. The estimated BMC (25) was used to obtain and further used to estimate lean mass from body weight and fat mass (refer Supplementary material).

### 2.2 Simulating micronutrient functionality

To the above-explained energetics framework, single and combined micronutrient effect were associated to growth outputs as per the established roles of each nutrient in growth (Supplementary Figure S1). To shortlist nutrients for supplementation in the virtual population, the prevalence of nutrient deficiencies in the Indian children was analyzed using national-level surveys such as CNNS and KHMU (4, 6). From these surveys, six micronutrients namely, iron, zinc, calcium, vitamin D, vitamin B9, and vitamin B12 were used in the model to determine their impact. Baseline micronutrient intake levels were estimated by referencing published studies on Indian children (27–31). These studies indicated that, at baseline, children were meeting only 35–60% of the RDA for the six key micronutrients.

To simulate a malnourished state, a set of correction factors for growth namely as “ϵ” (epsilon), “β” (beta), “η” (eta), and “λ” (lambda) were included in the model which were a function of % RDA intake of energy and micronutrients. These correction factors imposed a penalty on growth if the nutrient intake of the children satisfied < 75% of RDA. The correction factor “ϵ” was associated to % RDA intake of protein, zinc, vitamin B9 and vitamin B12. “ϵ” determined the FM and FFM distribution by influencing the distribution coefficient, “p” while the “β” was defined to estimate the BMC. %RDA intake of calcium and vitamin D were associated mainly with the BMC. To simulate dynamics of physical changes per month, calorie absorption efficiency defined as “η” was adjusted using a hill function, which estimated weight while a correction factor “λ” was adjusted for height changes respective to percentiles. The growth parameters “η” and “λ” were used to predict the influence of micronutrients on calorie absorption and height velocity, respectively. These parameters were linked to the %RDA intake of protein, zinc, and iron. In this way, the model was set to predict the effect of nutritional interventions, defined by their macronutrient (C, P, F) and micronutrient (Iron, Zinc, Calcium, Vitamin D, Vitamin B9, B12) composition, on anthropometric and body composition changes (height, weight, BMI, FM, FFM, and BMC). The results could also be categorized in terms of height and weight velocities, representing monthly changes in these parameters. All the parameters were obtained to fit the IAP data for boys and girls of the age group of 6–16 years covering 3rd−97th growth percentiles. A sample calculation is provided in section Modeling framework—Equations and Calculations of Supplementary material.

### 2.3 Biochemical profile modeling and dose-response predictions

To accurately simulate a population's biochemical profile, the present study utilized published prevalence data of micronutrient deficiencies in children and adolescents (4, 6), ensuring real-world variations in serum micronutrient levels were reflected. This data was employed to establish initial serum or urinary nutrient levels for the *in-silico* population, allowing for a realistic representation. The rate of change in serum micronutrient concentrations per unit dose, was influenced by baseline serum nutrient sufficiency levels. Published randomized controlled trials conducted in children were used to calculate these rates of change, considering both normal pre-intervention serum levels and cases with baseline deficiencies. This distinction allowed for an accurate assessment of the impact of supplementation in both healthy and deficient populations. For individuals with low baseline levels, the rate specific to the malnourished population was initially used. Post-supplementation, as serum levels increased and reached the 25th percentile of sufficiency, the rate of change associated with healthy individuals was applied, with further adjustment using Hill's equation at the 50th percentile to account for the saturation effect. Subsequently, the model was utilized to simulate the impact of varying product dosages on serum micronutrient levels for each child. This allowed for the prediction of how different doses would affect nutrient levels in both healthy and deficient populations, providing insights into optimal dosing strategies for targeted interventions.

### 2.4 Population characteristics

The virtual child population represented the 3rd−15th percentiles on the IAP 2015 growth charts, corresponding to z-scores between −1.03 and −1.88. A near-equal gender ratio (1:1) was maintained within the age groups. Caloric intake of the virtual population ranged from 829 to 1,850 kcal/day. A population of 2,000 individuals per age and gender was defined with specific height and weight ranges (Supplementary Table S1). Each experimental group hence had a population of 44,000 virtual children which accounts to a total study population of 308,000, as net cohort size of 7 experimental groups designed.

### 2.5 Intervention

The study designed seven distinct experimental setups to compare the isolated and combined effects of various dietary components on the study population (Table 1). Group 1 was the control receiving regular diet without milk. Group 2, group 3, group 5, and group 7 examined the effects of a combined macronutrient and micronutrient supplementation. Group 4 received milk alone, to assess its independent impact. While the group 2 and group 3 received the 1 serving and 2 servings ONS with water, respectively, group 5 and group 7 received the 2 serving ONS with milk. Group 6 consumed milk with just the macronutrient component of the ONS, helping to differentiate the effects of macronutrients from micronutrients and their potential interaction with milk. The difference between Groups 5 and 7 is the composition of the ONS provided. Group 5 received ONS-1, which contained the Horlicks-New composition, while Group 7 received ONS-2, the current Horlicks composition (referred as Horlicks henceforth). The nutrient compositions of ONSs are provided by Hindustan Unilever Ltd. Further details regarding the nutrient composition of the ONS, including macronutrient distribution as per AMDR (Acceptable macronutrient distribution ranges) guidelines, specific nutrient concentrations, and glycaemic index are available in the Supplementary material. As specified in Table 2, the macronutrient composition of ONS-1 and ONS-2 remains unchanged, with modifications made only to the micronutrient profile of ONS-1 as per one RDA compliance guidelines stated by the FSSAI (32). The nutrient composition of the milk used in this study was informed by data from IFCT 2017 and commercially available milk in India (33).

**Table 1:** Overview of experimental groups.

**Group label**	**Specification**	**Description**
Group 1	Control	Regular diet without milk or Horlicks supplementation.
Group 2	1 serve ONS in water	1 × 27 g daily serving of Horlicks-New in 150 mL water with regular diet.
Group 3	2 serves ONS in water	2 × 27 g daily serving of Horlicks-New in 150 mL water with regular diet.
Group 4	2 serves milk	2 × daily serving of 150 mL per serving of toned milk plus regular diet.
Group 5	2 serves ONS in milk	2 × 27 g daily serving of Horlicks-New in 150 mL per serving of toned milk with regular diet.
Group 6	2 serves CPF (macronutrients) in milk	2 × 27 g daily serving of Horlicks-New without micronutrients in 150 mL per serving of toned milk with regular diet.
Group 7	2 serves ONS-2 in milk	2 × 27 g daily serving of Horlicks in 150 mL per serving of toned milk with regular diet

**Table 2:** Nutrient composition of experimental interventions.

**Nutrients**	**Group 2**	**Group 3**	**Group 4**	**Group 5**	**Group 6**	**Group 7**
Energy (Kcal)	102	204	180	384	384	384
Carbohydrate (g)	21.33	42.66	14.4	57.06	57.06	57.06
Protein (g)	2.97	5.94	9.3	15.24	15.24	15.24
Fat (g)	0.54	1.08	9.3	10.38	10.38	10.38
Vitamin D (mcg)	7.425	14.85	3.66	18.51	3.66	8.66
Vitamin B9 (mcg)	67.5	135	21.09	156.09	21.09	220.89
Vitamin B12 (mcg)	1.08	2.16	1.62	3.78	1.62	2.62
Iron (mg)	7.02	14.04	0.45	14.49	0.45	14.45
Calcium (mg)	200.07	400.14	354	754.14	354	754
Zinc (mg)	2.241	4.482	0.99	5.472	0.99	5.49

Group 2: 1 serve Horlicks-New; Group 3: 2 serve Horlicks-New; Group 4: Plain milk; Group 5: Horlicks-New with milk, Group 6: Isolated macronutrients of Horlicks-New with milk, Group 7: Horlicks in milk.

### 2.6 Statistical analysis

The core energy balance computations, *in-silico* population generation and model simulations were performed in the MATLAB software. The details are described in the Supplementary material (Pages 5–8); which includes all equations and calculations. Statistical analysis and graphical visualization of the results were done using Python (version 3.12.3). *P*-value of < 0.05 was used to evaluate significance between two groups. Comparisons of parameters between experimental groups were performed using the independent *t*-test. Cohen's D was used to quantify the magnitude of the difference between experimental groups. Cohen's d thresholds of < 0.2 indicates a small effect, 0.2–0.5 indicates a medium effect, and >0.5 indicates a large effect (34).

## 3 Results

### 3.1 Model benchmarking

Parameter values in the present model were estimated to trace various percentiles of height and weight for boys and girls between the ages of 6–16 years. For each age and gender, the BMR and TEF was estimated based on body weight, caloric intake and PAL. TEF was estimated as per defined amounts of carbohydrate, protein, and fat (35). Supplementary Figure S2 shows the illustrative trends of 3rd, 10th, and 50th percentiles for various ages for both boys (Supplementary Figure S2A) and girls (Supplementary Figure S2B). The model with the set parameter could trace the growth trajectory for various percentiles with < 10 and < 3% error for height and weight, respectively. The model was therefore considered a platform to assess the percentile of a child and can estimate the caloric intake for a specific age and gender.

### 3.2 Model validation

**Figure 2:**
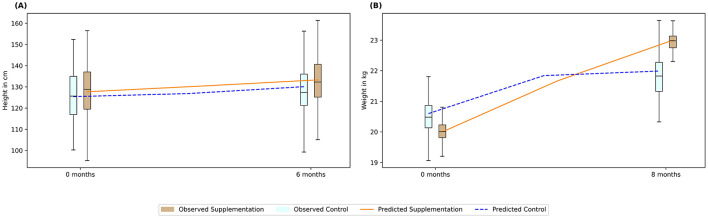
Validating predictions of intervention-led anthropometric changes using published studies. The bar plots represent data from the sample studies, while the lines indicate predicted values. **(A)** Zinc supplementation; **(B)** Protein supplementation. Box: interquartile range; top, middle, and bottom edge of the box: Q1, Q2, Q3 respectively. Blue bars: Control; Brown bars: Intervention; Blue dotted line: Prediction for control; Orange solid line: Prediction for intervention.

The model was validated to predict the height and weight change due to an intervention. Example cases of zinc and protein supplementation are illustrated in Figures 2A, B, respectively. A study by Rerksuppaphol and Rerksuppaphol (36) was used as a sample study to validate intervention effects of zinc supplementation on zinc deficient children of 6–10 years. With 15 mg of zinc supplementation for 6 months, the model could predict marginal increase in height, as also observed in the study. This is because the supplementation could satisfy more than 75% RDA intake for zinc which removed the growth penalty on “ε”, “η”, and “λ” as mentioned earlier. While the correction factor “λ” could increase the height gain with zinc, the penalty from other associated micronutrients remained in the model. This restricted the optimal height increase for the population and hence a marginal increase was observed.

Similarly, a sample case study was used for assessing protein supplementation effect (37) reporting an intervention of 20 g of skim milk powder for 5 days a week for 8 months in 7- to 13-year-old children. Figure 2B illustrates that the model was able to capture the weight increase by 1.15-fold due to protein supplementation, while the cohort with no supplementation (i.e., control group) increased only by 1.06-fold. Protein deficiency is also associated with lower macro-nutrient absorption and lower FFM. The increase in weight in the cohort with no supplementation saturated beyond 4 months, in contrast, the cohort submitted with supplementation continued to have growth results until 8 months. This was associated with more than 75% RDA of protein being satisfied due to supplementation unlike in the control population.

**Figure 3:**
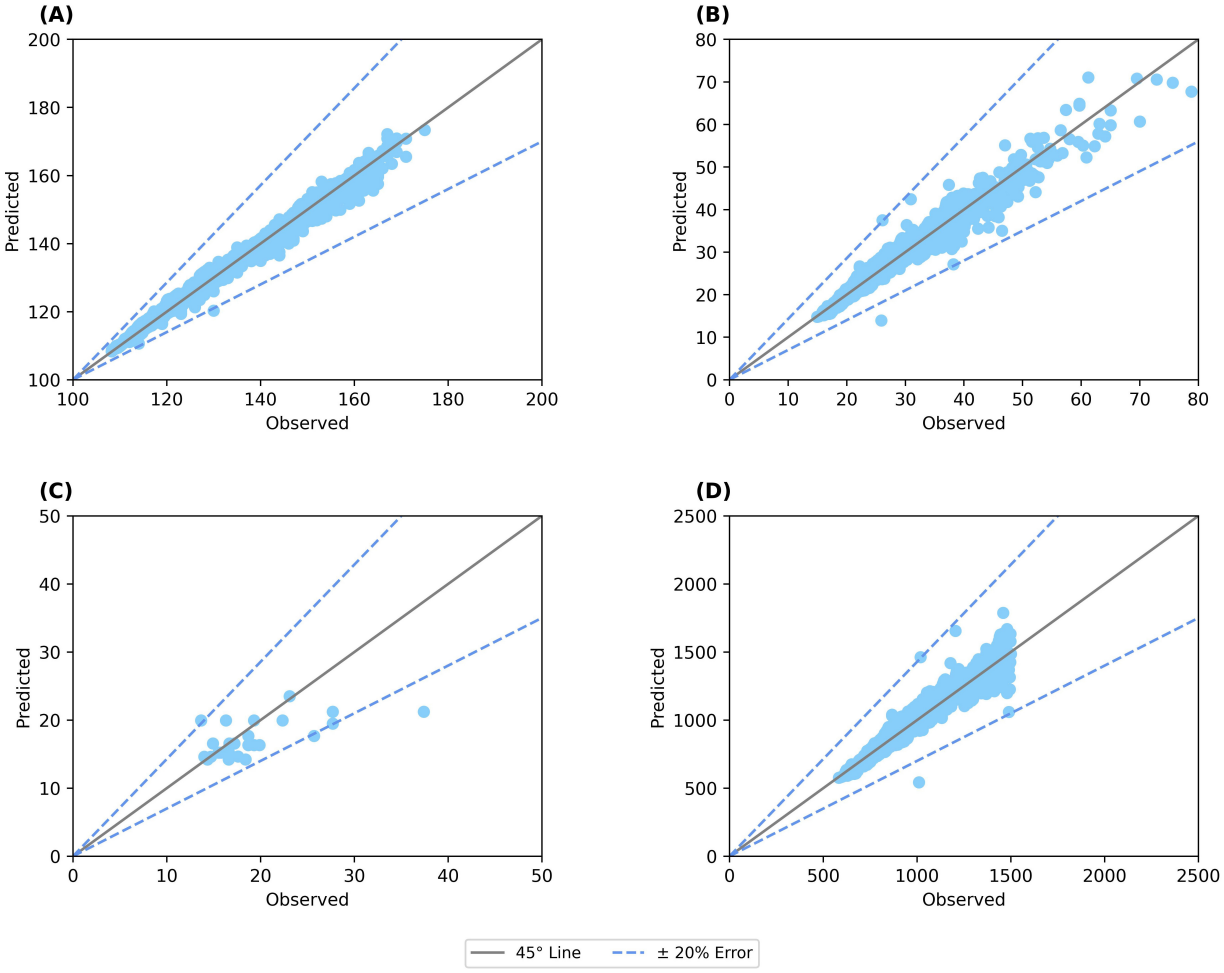
Validation of anthropometric and body composition parameters against clinical data. **(A)** Predicted height; **(B)** Predicted weight; **(C)** Body fat percentage; **(D)** BMC (bone mineral content). Straight line: 45° line; Dotted line: Error percentage.

The model was further used to predict the height and weight of 3,916 children monitored over a year (38). Figure 3A shows the predicted height plotted against the observed height, showing an error < 5%, while Figure 3B shows the graph for weight indicating an error < 10%. Similarly, the model was also able to predict the body composition with an error < 20% for body fat (Figure 3C) and < 10% for BMC for 3,694 children (Figure 3D). Thus, the model was validated to represent the growth percentiles and predicted body composition of children between 6 and 16 years accounting for both the macro and micro-nutrients.

Apart from predicting growth percentiles, the model could also predict a specific intervention effect over a specific dosage and duration. Supplementary Figure S3, Case 1a and S3 Case 1b shows the effect of 100 kcal intervention above the basal diet for a 7-year boy in the 10th percentile of weight. The model predicts that the intervention may result in 4 cm growth in height over a year and 2.4 kg growth in weight (Supplementary Figure S3, Case1b). The growth was only due to the macronutrient (66% carbohydrate, 24% fat, and 8% protein) as the effect of micronutrients was not accounted for in the model prediction. This results in a height and weight velocities of 0.34 cm/month and 202 g/month, which is a reasonable growth as obtained from the IAP data.

Similarly, protein supplementation (Supplementary Figure S3, Case 2a; Supplementary Figure S3, Case 2b) accounting for 12% of total calories was administered to girls aged 12 years in the 10th percentile for height along with 100% RDA intake of micronutrients, would result in a height growth of 2.5 cm and a weight gain of 2.5 kg over a year. This translates to a height velocity of 0.21 cm/month and a weight velocity of 200 g/month, respectively, showing a percent shift of 1.79% in height and 8.1% in weight. Thus, the model in principle can be used to monitor the growth trajectory of children and predict the possible growth outcomes due to an intervention of macro and micronutrient supplementation.

### 3.3 Impact of ONS intervention on intakes of key micronutrients

**Figure 4:**
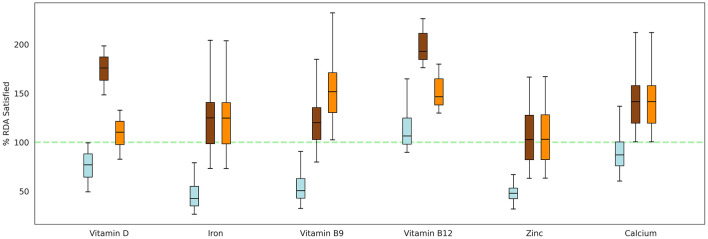
Percentage of RDA satisfied for key micronutrients by both the ONS formulations, given in addition to the basal diet. Box: interquartile range; top, middle, and bottom edge of the box: Q1, Q2, Q3, respectively. Blue: Group 4 (Plain milk); Brown: Group 5 (Horlicks-New); Orange: Group 7 (Horlicks).

To comprehensively evaluate the impact of both the ONSs on %RDA satisfaction, Groups 5 (Horlicks-New) and 7 (Horlicks) were administered their respective ONS with milk interventions, in addition to their baseline caloric intake (Table 1). These groups were then compared to the plain milk group (Group 4) to assess the relative effectiveness of each intervention. At the baseline, diet of these children was assumed to be sufficing 35% to 60% RDA of the six micronutrients. Post intervention, the plain milk group (Group 4) was insufficient to meet the RDA requirements for most micronutrients, except for vitamin B12, where intake exceeded 100% RDA. Notably, for key micronutrients such as zinc, iron, and vitamin B9, the mean intake remained below 75% of the RDA. In contrast, addition of Horlicks to milk (Groups 5 and 7), could lift the mean of the population above 100% RDA intake for all six micronutrients (Figure 4). In terms of zinc adequacy, while the population mean achieved 100% RDA, the third quartile of participants satisfied between 75 and 100% RDA. Despite the differing dosages of vitamin D, vitamin B9, and vitamin B12 between the two ONSs, both interventions could satisfy RDA for these vitamins. Similarly, energy could satisfy more than 75% RDA in the population, whereas protein could contribute up to 10–12% of total energy by the end of the intervention with ONS with milk.

### 3.4 Impact of ONS intervention on biochemical profile

The biochemical platform, developed using the aforementioned methodology, is validated in Supplementary Figure S4 based on published nutrient supplementation studies in children. The average error percentage across all parameters, including zinc, ferritin, B-complex vitamins, calcium, and vitamin D, was 7.69%. The platform was then used to predict biochemical changes across different intervention scenarios, focusing on the comparison between milk alone and Horlicks with milk. For this analysis, Groups 4 (plain milk group), 5 (Horlicks-New group), and 7 (Horlicks group) were compared to assess their respective impacts on biochemical profile.

As shown in Supplementary Figure S5A, majority of the population had serum calcium levels below the deficiency threshold at baseline. Intervention with both the Horlicks compositions with milk (groups 5 and 7) effectively shifted the mean serum calcium levels above the deficiency cut-off by the 8th month, while the plain milk group (group 4) reached the cut-off at the 12th month. Supplementary Figure S5B illustrates a steady increase in serum ferritin levels with both the Horlicks interventions followed by a physiological saturation. Specifically, serum ferritin levels increased by an average of 48% from baseline in Group 5 by the 8th month, with a further 10% increase over the next 4 months, suggesting a plateau effect in absorption. On the contrary, the plain milk group showed no significant change in serum ferritin. Similarly, as depicted in Supplementary Figure S5C, serum zinc levels showed a 14% average increase up to the 8th month in group 5, followed by an additional 4% increase over the subsequent 4 months. For parameters such as serum vitamin D, serum vitamin B9, and serum vitamin B12, the plain milk intervention resulted in an average increase of 60%, 7%, and 80%, respectively, by the 12th month compared to baseline. In contrast, children in Group 5 who consumed Horlicks-New demonstrated equivalent or greater improvements by the 4th month, with serum vitamin D increasing by 97%, serum vitamin B9 by 15%, and serum vitamin B12 by 76%.

By the end of the 12-month intervention, 100% of the population in the Horlicks intervention groups (Groups 5 and 7) had biochemical parameters above the 50th percentile sufficiency range, except for serum calcium. For serum calcium, most participants remained within the 25th−50th percentile sufficiency range. Statistically significant differences in biochemical parameters were observed in Group 5 compared to the milk group as early as the 4th month (Supplementary Table S3). When comparing Group 5 and Group 7, vitamin B12 and vitamin D showed large effect sizes, with Cohen's D values of 4.9 and 3.6, respectively, at 12 months, attributed to the different dosages.

### 3.5 Dose-response impact of ONS intervention on anthropometric outcomes

**Figure 5:**
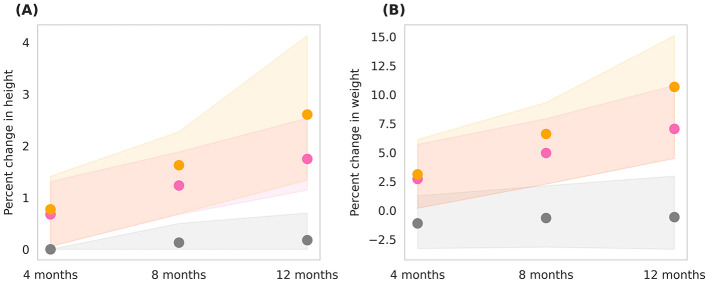
Post-intervention changes in anthropometric parameters among Group 1 (Control), Group 2 (1 serve Horlicks-New), and Group 3 (2 serves Horlicks-New). **(A)** Percent change in height. **(B)** Percent change in weight. Gray: Group 1 (Control); Pink: Group 2 (1 serve Horlicks-New); Orange: Group 3 (2 serves Horlicks-New).

The *in-silico* cohort simulated the effects of Horlicks-New dosages by comparing Group 2 and Group 3 (refer to Tables 1, 2). Figure 5 shows the post-intervention percent change in height and weight and population distribution as standard error, shown in shaded region for each group. It is clear from the figure that for children between 3rd and 15th percentile of height and weight, the basal diet was not sufficient for healthy growth, with a marginal drop in mean weight and no significant change in height. However, with the intervention of one serving of Horlicks-New with water (Group 2), there was a 1.5% increase in height and a 6% increase in weight from the baseline values. In contrast, providing two servings of Horlicks-New in water (Group 3) resulted in a 2.6% increase in height and a 10% increase in weight.

When analyzing growth velocities, by the end of intervention, Group 2 demonstrated an average height velocity of 0.2 cm/month and a weight velocity of 134 g/month. In comparison, Group 3 showed higher growth velocities, with an average height velocity of 0.26 cm/month and a weight velocity of 203 g/month. Cohen's D values indicated small to medium effect sizes at the 4th and 8th months of the intervention (Table 3). By the 12th month, however, the difference between the two groups became statistically significant (*p* < 0.001) with a large effect size (Cohen's D = 4), reflecting a substantial impact of the intervention over time. Greater improvements in group 3 in comparison to group 2 indicates that 1 serving did not provide saturation in effect and 2 servings Horlicks-New can be recommended for optimum growth results in the population.

**Table 3:** Statistical comparison of between-group anthropometric outcomes across timepoints.

		**4 months**			**8 months**			**12 months**		
		***t*** **-stats**	***p*** **-value**	**Cohen's D**	***t*** **-stats**	***p*** **-value**	**Cohen's D**	***t*** **-stats**	***p*** **-value**	**Cohen's D**
Group 3 vs. Group 2	Height	1	0.461	0.3	2	0.021	0.9^*^	3	0.003	1.3^*^
	Weight	1	0.474	0.3	2	0.02	0.9^*^	4	0.001	1.5^*^
	BMI	1	0.545	0.2	2	0.033	0.9^*^	4	0.001	1.4^*^
	Fat free mass	1	0.345	0.4	3	0.013	1^*^	4	< 0.0001	1.6^*^
	Fat mass	0	0.674	0.2	1	0.555	0.2	1	0.155	0.6
	BMC	0	0.686	0.2	2	0.05	0.8^*^	2	0.051	0.8^*^
Group 5 vs. Group 4	Height	7	< 0.0001	2.5^*^	8	< 0.0001	3.1^*^	7	< 0.0001	2.6^*^
	Weight	7	< 0.0001	2.5^*^	9	< 0.0001	3.2^*^	9	< 0.0001	3.3^*^
	BMI	6	< 0.0001	2.5^*^	8	< 0.0001	3.1^*^	7	< 0.0001	2.7^*^
	Fat free mass	6	< 0.0001	2.4^*^	7	< 0.0001	2.8^*^	8	< 0.0001	3.2^*^
	Fat mass	6	< 0.0001	2.3^*^	5	< 0.0001	2^*^	4	< 0.0001	1.6^*^
	BMC	5	< 0.0001	2.1^*^	8	< 0.0001	3.1^*^	6	< 0.0001	2.3^*^
Group 5 vs. Group 6	Height	5	< 0.0001	2^*^	3	0.002	1.3^*^	2	0.072	0.7
	Weight	5	< 0.0001	2.1^*^	4	0.001	1.4^*^	2	0.022	0.9^*^
	BMI	6	< 0.0001	2.2^*^	4	0.001	1.4^*^	2	0.043	0.8^*^
	Fat free mass	6	< 0.0001	2.1^*^	4	0.001	1.4^*^	3	0.009	1.1^*^
	Fat mass	5	< 0.0001	1.9^*^	1	0.164	0.5	0	0.854	0.1
	BMC	5	< 0.0001	1.9^*^	4	0.002	1.3^*^	1	0.187	0.5

^*^Large effect size.

### 3.6 Impact of plain milk vs. ONS with milk intervention

**Figure 6:**
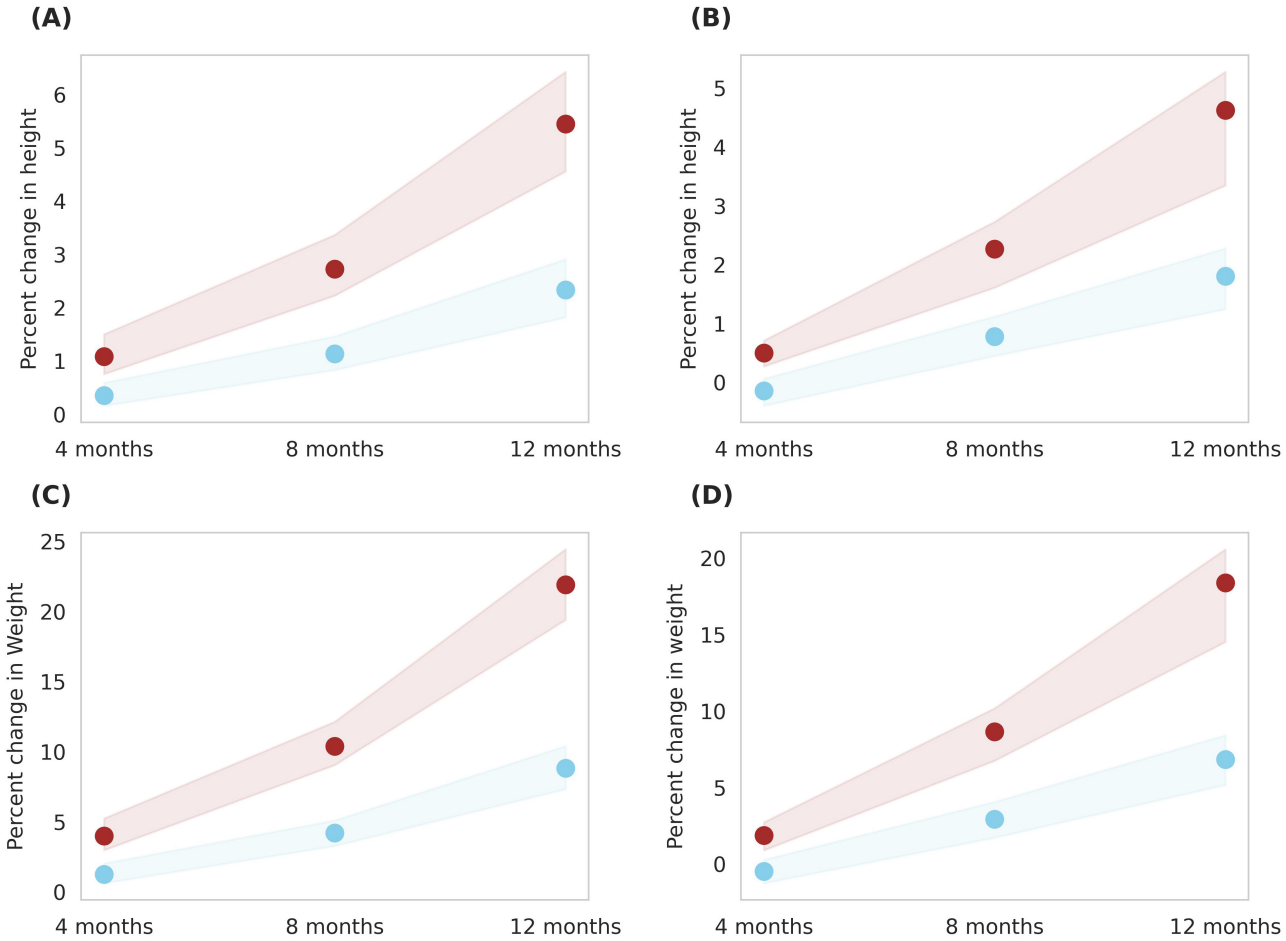
Gender-wise depiction of post-intervention change in height and weight among Group 4 (Plain milk) and Group 5 (Horlicks-New) children aged 6–9 years. **(A)** Height change in girls; **(B)** Height change in boys; **(C)** Weight change in girls; **(D)** Weight change in boys. Blue: Group 4 (Plain milk); Brown: Group 5 (Horlicks-New).

**Figure 7:**
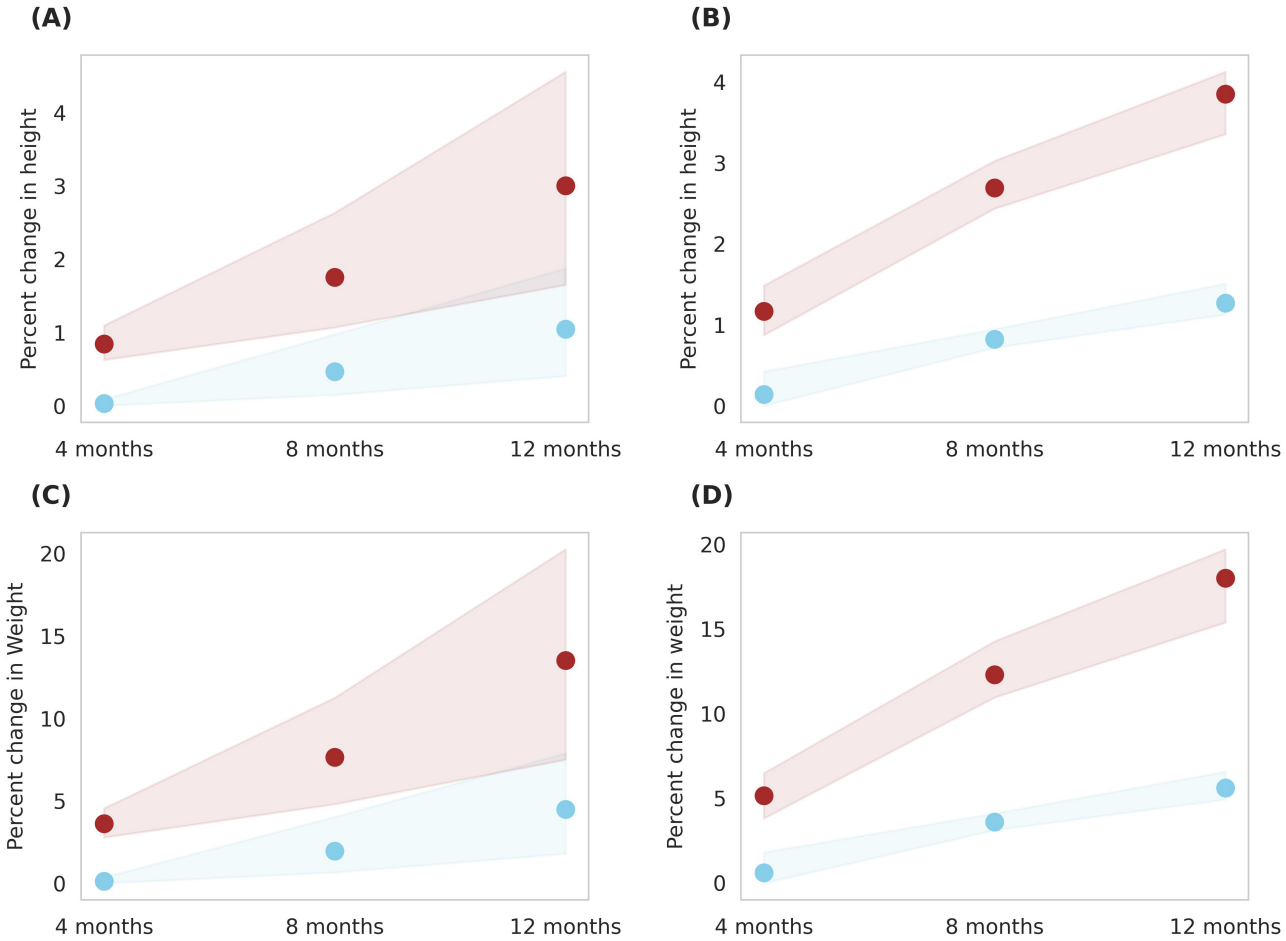
Gender-wise depiction of post-intervention change in height and weight among Group 4 (Plain milk) and Group 5 (Horlicks-New) children aged 10 to 16 years. **(A)** Height change in girls; **(B)** Height change in boys; **(C)** Weight change in girls; **(D)** Weight change in boys. Blue: Group 4 (Plain milk); Brown: Group 5 (Horlicks-New).

Simulations were conducted to compare the effects of two servings of milk (Group 4) and two servings of Horlicks-New with milk (Group 5) on a population stratified by sex and age. The analysis was performed separately for boys and girls within the 6–9 and 10–16 age groups (Figures 6, 7).

As illustrated in Figure 6, 6- to 9-year-old children in group 4 could achieve an average 1% increment in height in both the genders while group 5 boys and girls improved by 5.2 and 4.5%, respectively, in 12 months. This change was statistically significant (*p* < 0.001) with an effect size of 4.2 and 4.0 for boys and girls, respectively. Children aged 10–16 years showed a post-intervention height increase of 2.8% in girls and 3.8% in boys (Figure 7). These improvements were significantly greater (*p* < 0.001) than those in Group 4, where girls and boys experienced height gains of 1.04 and 1.26%, respectively. The height increments yielded large effect sizes, with Cohen's D values of 7.6 for boys and 1.7 for girls. The height velocities were in the range of 0.2–0.56 cm/month for the entire population of 6–16 years children while the rate of growth was higher in the latter half of the year (i.e., 8–12 months) in Group 5 (Horlicks-New with milk).

Similar results were observed in weight, wherein, milk intervention (group 4) achieved an average weight increase of 7.8% for both ages and gender. With Horlicks-New with milk intervention (group 5), the percentage weight change in girls were 22 and 13% for the age group 6–9 and 10–16 years, respectively, while the boys in group 5 showed 18% increase in weight for both ages (Figures 6, 7). These increments achieved a statistical significance (*p* < 0.001) when compared to group 4.

On comparison between age groups, the younger age had a higher percent growth than the older children as the net calories and micronutrient intervention was the same to the entire population. Also, the percent growth change with Horlicks-New with milk was 2.6 and 11.5% higher for height and weight, respectively, compared to plain milk intervention.

**Figure 8:**
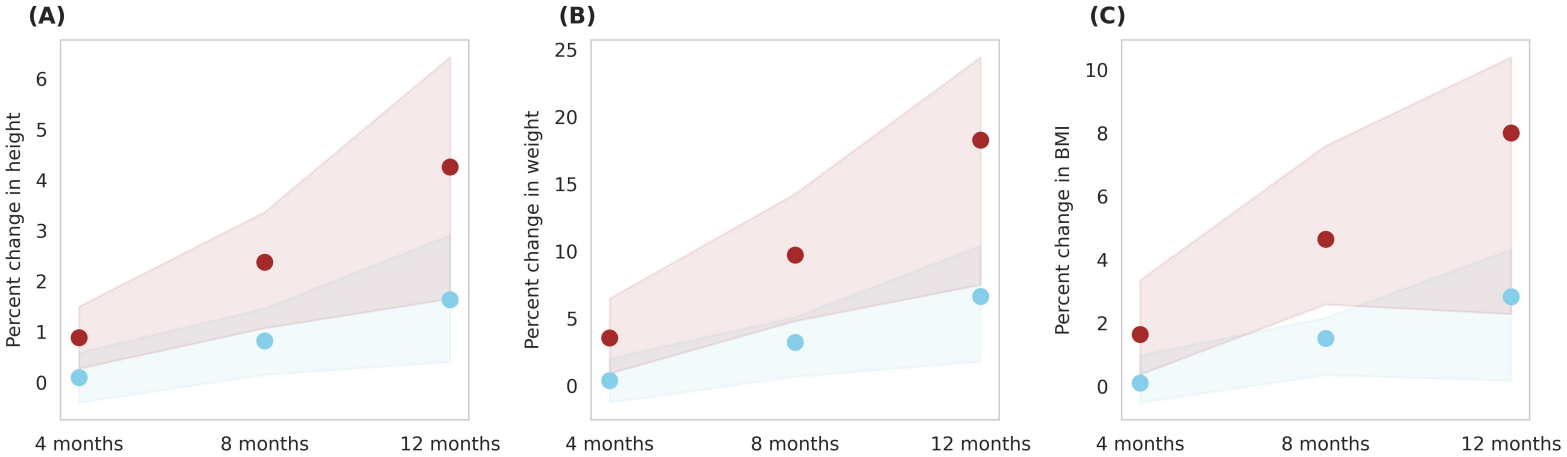
Post-intervention changes in anthropometric parameters among Group 4 (Plain milk) and Group 5 (Horlicks-New). **(A)** Percent change in height. **(B)** Percent change in weight. **(C)** Percent change in body mass index (BMI). Blue: Group 4 (Plain milk); Brown: Group 5 (Horlicks-New).

The intervention's effect was also analyzed across the entire *in-silico* population, encompassing both genders and covering the 6–16 age range (Figure 8). An overall percent height change and percent weight change of 4.3 ± 1.3% and 18.3 ± 4.5%, respectively, was observed with Horlicks-New with milk intervention (group 5). This resulted in a net percent BMI change of 8 ± 2.4%. Intervention with milk alone (group 4) observed change of 2.8 ± 1.2% change in BMI.

**Figure 9:**
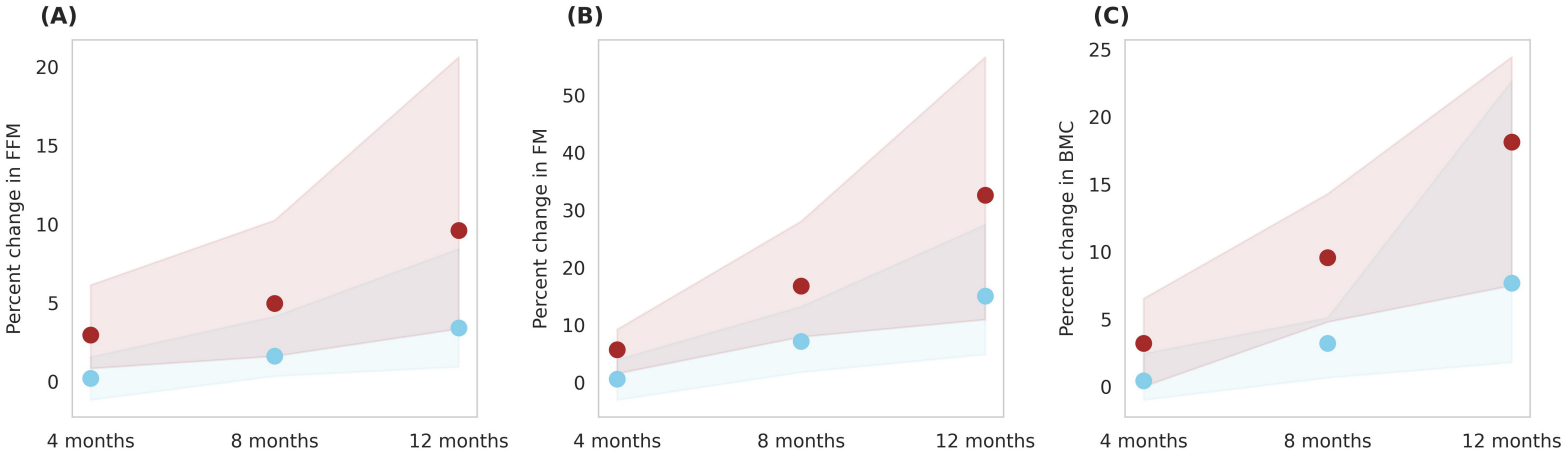
Post-intervention changes in body composition parameters among Group 4 (Plain milk) and Group 5 (Horlicks-New). **(A)** Percent change in FFM (fat-free mass). **(B)** Percent change in FM (fat mass). **(C)** Percent change in BMC (bone mineral content). Blue: Group 4 (Plain milk); Brown: Group 5 (Horlicks-New).

Figure 9 illustrates prediction of body composition changes with Horlicks-New intervention (group 5) in overall population. The analysis indicated 8% increase in FFM and a 30% increase in FM by the end of intervention. In terms of absolute mass, FFM increase by 3.18 kg and FM increased by 0.85 kg, indicating a ~78% of weight gain from FFM. Also, it can be noted that the Horlicks-New with milk resulted in 1.10 and 1.14-fold higher in FFM and FM, respectively. The intervention of milk alone (group 4) resulted in 6% increase in BMC while the Horlicks-New with milk showed an increase of 18.13 ± 4.4%. The observed increases were statistically significant (*p* < 0.0001) and demonstrated a large effect size, as indicated in Table 3. Thus, the intervention of Horlicks-New with milk could overall benefit the population in 3rd−15th percentile across gender and age.

### 3.7 Impact of ONS intervention with and without added micronutrients

**Figure 10:**
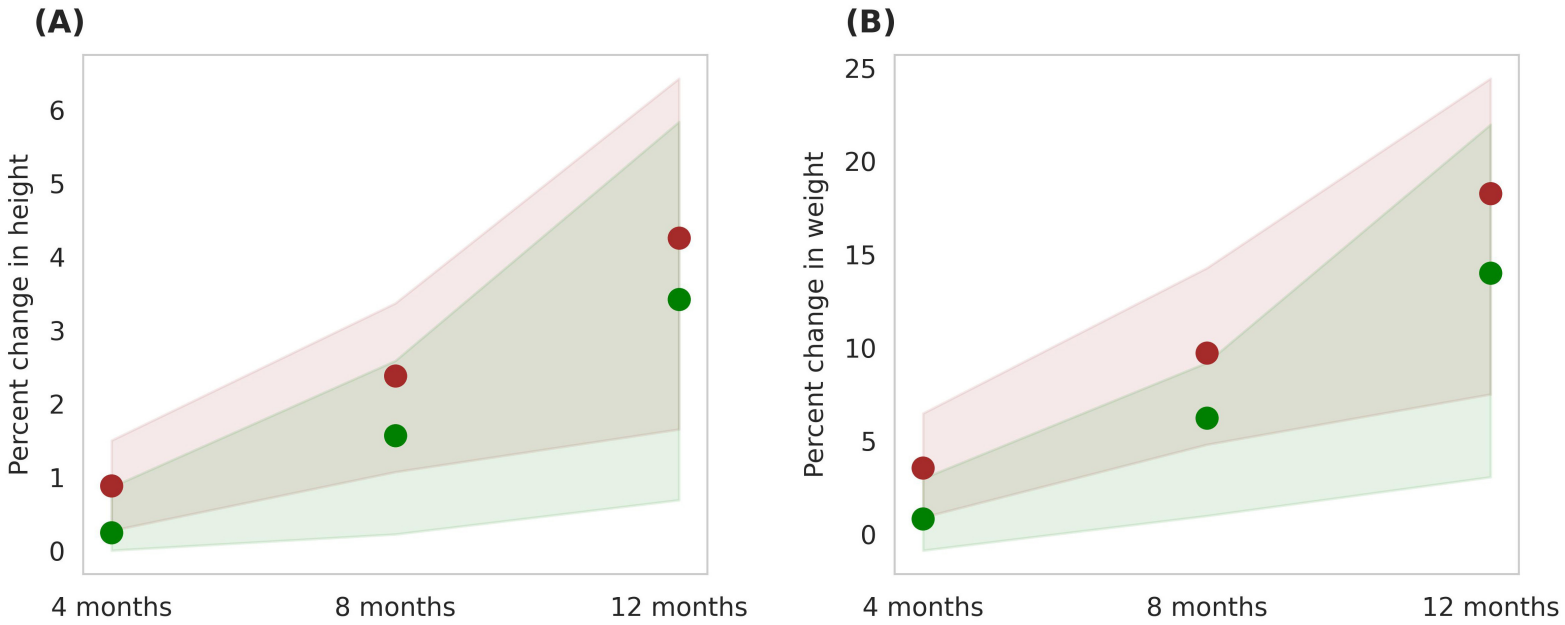
Post-intervention changes in anthropometric parameters among Group 5 (Horlicks-New) and Group 6 (Macronutrient-only). **(A)** Percent change in height. **(B)** Percent change in weight. Brown: Group 5 (Horlicks-New); Green: Group 6 (Macronutrient-only).

The Horlicks-New composition contains both macronutrients and micronutrients (Tables 1, 2) and to differentiate the effect of micronutrients, an intervention protocol was simulated wherein only macronutrients within the Horlicks composition were considered. Figure 10 presents a comparison between the macronutrient-only ONS group (Group 6) and the Horlicks-New with milk group (Group 5). Post-intervention, Group 6 demonstrated a height increase of 3.4 ± 1.3% and a weight increase of 14 ± 4.8%. In contrast, Group 5, which received both macronutrients and micronutrients, showed an additional increase in height and weight of 0.92 ± 0.23% and 4.3 ± 1.29%, respectively, compared to Group 6. Thus, the overall change observed with Horlicks with milk intervention is the sum effect of macronutrients and micronutrients.

Furthermore, Group 7, which received the Horlicks intervention, was also analyzed for its impact on anthropometric and body composition parameters. When compared, both the Horlicks interventions produced similar outcomes, resulting in statistically insignificant differences between Group 5 and Group 7 in terms of fat-free mass (FFM, *p* = 0.4) and bone mineral content (BMC, *p* = 0.79). This similarity in results is likely due to both ONSs meeting more than 75% of the RDA for the six key micronutrients, ensuring adequate nutrient intake across both groups. The results for group 7 are presented in Supplementary Table S2 and Supplementary Figure S6.

## 4 Discussion

The developed modeling framework accounted for the growth parameters for boys and girls in the age range of 6–16 years to match various percentiles. The framework demonstrated the capability to predict the growth percentiles as represented in the IAP growth chart. The study benchmarks the effect of macronutrients on net calorie intake and the impact of key micronutrients known to be limiting in the Indian child population. The model explicitly accounted for the effects of protein and key micronutrients on overall child growth by integrating both anthropometric measurements (height and weight) and body composition metrics (FFM, FM, and BMC). Further, the effect of micro and macronutrients were integrated to bring out a predictive platform for child growth. In the present study, capability of the model to simulate the effect of an intervention on a child was demonstrated and further extended to see an effect on a population thereby simulating an *in-silico* trial. Specifically, the study assessed the effect of Horlicks-New and Horlicks intervention on the physical biochemical profile and growth of Indian children.

Among the seven experimental groups, the Horlicks interventions (Groups 5 and 7) had the most pronounced effect on both biochemical profiles and physical growth. Adequate intakes of protein, zinc, and iron improved deficient serum levels, leading to significant increases in height velocity, weight velocity, and BMI. The combined intake of these nutrients in Groups 5 and 7 effectively enhanced both serum levels and growth outcomes, consistent with clinical evidence supporting multi-nutrient interventions (16, 39). The mean height percentile increased from the 10th to the 11th, while the mean weight percentile rose from the 10th to the 17th. Post-intervention, the population mean of 10th percentile height exhibited an average height velocity of 0.42 cm at the 12th month; which as per IAP 2015 data is a height velocity of children in 25th−75th percentile range; indicating that the intervention had a clinically meaningful impact. Group 5 (Horlicks-New) also demonstrated significant improvements in FFM and BMC, outperforming both the plain milk group (Group 4) and the group receiving macronutrient supplementation with milk (Group 6). These gains were driven by the combined intake of protein, calcium, vitamin D, and zinc, which improved serum nutrient levels and contributed to the observed increases in FFM and BMC (40–42). Group 7, which received the Horlicks intervention, demonstrated comparable efficacy to Group 5, as both ONSs provided sufficient intake of all key micronutrients. Consequently, both the Horlicks interventions demonstrated significant improvements across all measured outcome parameters post-intervention.

With the plain milk intervention (150 mL twice daily), the % RDA increases for protein, zinc, and iron were calculated to be 33%, 12%, and 2.5%, respectively, leading to model-predicted height and weight velocities of 0.17 cm/month and 118 g/month. These predictions are in close agreement with the findings of Grillenberger et al., who observed height and weight velocities of 0.17 cm/month and 70 g/month, respectively, in a cohort of children with a mean age of 7.31 years after a similar milk intervention (350 mL daily) (43). The alignment between the model's predicted growth velocities and the clinical trial outcomes supports the validity of the modeling framework in simulating growth patterns resulting from milk supplementation.

Building on these results, the Horlicks-New with milk (Group 5) was able to improve the % RDA of key growth nutrients—protein by 54%, zinc by 70%, and iron by 80%—which effectively contributed to the observed improvements in growth metrics. This intervention resulted in higher height and weight velocities of 0.44 cm/month and 337 g/month, respectively. The predicted outcomes for the group 5 closely aligned with those reported in clinical trials. For instance, the model's net prediction of a 5.3 cm increase in height and 4 kg weight gain over a 1-year intervention is comparable to a study by Thomas et al. (44), which found an average height increase of 6 cm and a weight gain of 3.65 kg in prepubertal children receiving a similar intervention. Additionally, an Indian study from 2,011 observed a height velocity of 0.5 cm/month and a weight velocity of 225 g/month following 4 months of fortified beverage supplementation (45). In comparison, the model predicted a height velocity of 0.4 cm/month and a weight velocity of 211 g/month for a similar 4-month intervention with ONS in milk. These findings demonstrate the model's capability to accurately replicate the outcomes observed in clinical trials following ONS supplementation.

To further analyze the specific nutrient contributions, the study explored how macronutrients and micronutrients individually affected growth outcomes. To distinguish the impact of micronutrients in the Horlicks intervention, post-intervention effects of group 5 and group 6 were compared. The simulation estimated that administering macronutrient-rich ONS with milk, a 384-kcal intervention, over 4 months, would result in height and weight increases of 0.19–0.30 cm and 0.17–0.18 kg, respectively, in group 6. This supplementation led to an average shift in growth percentiles from the 10th to the 13th. For comparison, a study by Nawab et al. involving 7-year-old children showed height gains of 0.1–0.8 cm and weight increases of 0.1–1 kg with a 535-kcal macronutrient intervention over a similar duration (46).

The observed difference in the growth velocities for groups with and without micronutrients underscores the added impact of micronutrients. The Horlicks intervention comprising both macronutrients and micronutrients when administered with milk (group 5), resulted in an additional average increase of 1.18 cm in height and 1.05 kg in weight over a 1-year period, compared to the group receiving only the equivalent macronutrients through ONS in milk (group 6). These findings clearly demonstrate that the inclusion of micronutrients enhanced caloric absorption and facilitated healthier gains in FFM, FM, and BMC which subsequently led to more pronounced improvements in height and weight. The greater percentage change in FFM observed in group 5 compared to group 6, as predicted by the current study, is consistent with findings from previously published trials (38, 47). This underscores the model's reliability and the practical applications of its use.

To further explore the impact of Horlicks dosage, a comparison between the two serving sizes (Group 3 vs. Group 2) revealed enhanced efficacy with two servings of Horlicks compared to a single serving, suggesting that the intervention's benefits continued to increase without reaching a saturation point within 12 months. This observation suggests the potential for extending the intervention beyond 12 months to maximize benefits, as saturation was not achieved during the observed timeframe.

To compare the gender and age-specific alignment with previous studies, the original dataset received from Vijayalakshmi et al. (38) was used. As per this dataset, the 7–9 years age group, observed a height velocity of 0.59 cm/month for boys and 0.62 cm/month for girls, with a nutrient intervention of 453 kcal. In comparison, our model predicted a height velocity of 0.44 cm/month for boys and 0.46 cm/month for girls in the Horlicks-new with milk group (384 kcal intervention). Notably, the trial observed a relatively higher height velocity which is consistent with the findings captured in our model. Additionally, in the 10–12 years age group, the trial observed a height velocity of 0.62 cm/month for boys and 0.58 cm/month for girls, compared to the predicted velocities of 0.35 cm/month for boys and 0.32 cm/month for girls. Boys in this age group showed a greater height velocity, consistent with the model predictions. Overall, no significant differences in growth outcomes were observed between age groups in both the study and the model predictions.

In terms of intervention duration, the present analysis indicated that Horlicks with water require a minimum of an 8-month intervention to exhibit statistical significance compared to the control group. Conversely, Horlicks with milk intervention demonstrated significant improvements in comparison to milk as early as the 4th month, with the maximum impact observed by the 12th month. This is because the inclusion of milk alone was insufficient to meet both the macronutrient and micronutrient requirements for these children, particularly due to the inadequacies of the basal diet of children belonging to 3rd to 15th growth percentiles. Addition of Horlicks to milk improved nutrient intake and ensured that a higher percentage of their RDA for key micronutrients was achieved. This effectively improved serum nutrient levels, consequently supporting optimal growth.

This manuscript demonstrates the strength of *in-silico* modeling as a highly efficient tool for predicting the effects of nutritional interventions, particularly in child populations where large-scale clinical trials may be challenging. The model's ability to integrate various datasets and simulate complex growth trajectories highlights its utility in optimizing intervention strategies. Additionally, its cost-effectiveness and faster timeline compared to traditional trials are significant strengths, allowing for a broader application in resource-constrained settings. However, the study does have certain limitations that must be acknowledged. While the model is best suited for population-level data analysis and hypothesis generation, modeling outliers or edge cases may present higher error margins. Although the model is benchmarked against population data with height and weight percentiles, enabling accurate predictions of an intervention's impact on populations within specific percentiles, it requires further validation for other population distributions, such as different ethnic groups. The approach can be generalized by recalibrating the model parameters for diverse populations, but this step must be completed before the model can be used for broader predictions. Additionally, the model operates under the assumption of consistent nutrient absorption and utilization across the population, primarily considering macro and micronutrients. However, analyzing the intervention effect in populations affected by factors like clinical malnutrition or specific diseases (48) may be challenging, as the model may not accurately capture outcomes without further benchmarking and validation. Despite these constraints, the model demonstrated acceptable prediction accuracy, with validation results showing robust initial assumptions.

## 5 Conclusion

The findings of this study underscore the effectiveness of model-based validation in predicting the impact of ONS interventions on child growth. The Horlicks with milk intervention for children in the 3rd to 15th percentile resulted in significant improvements in serum nutrient levels and key growth parameters, attributed to its nutrient-rich composition. Improved serum levels resulted in greater monthly gains in FFM and BMC, contributing to healthier weight gain. Additionally, the Horlicks-New intervention demonstrated superior height velocities, further supporting its role in promoting optimal growth outcomes. Analysis of differential contribution of micronutrients revealed that the effects of the Horlicks intervention were due to the combined contributions of both macronutrients and micronutrients in its composition. A clear dose-response effect was observed, with two servings of Horlicks with milk yielding significantly better outcomes than one serving. Moreover, Horlicks with milk outperformed intervention of milk alone, with notable effects emerging as early as the 4th and 8th months of intervention. However, applying these study findings to other ethnicities requires further validation, which future research can address. The current phenomenological model can be extended to assess cost-effectiveness and buying capacity, providing a more comprehensive economic perspective for future analyses. These findings serve as a preliminary impact assessment and provide a foundation for future clinical trials, supporting the development of targeted nutritional strategies to enhance child growth outcomes.

## Data Availability

Publicly available datasets were analyzed in this study. This data can be found here: https://indianpediatrics.net/jan2015/jan-47-55.htm; https://nhm.gov.in/WriteReadData/l892s/1405796031571201348.pdf.
