# Supplementary material for: Evaluating the impact of an oral nutrition supplement on biochemical profile, growth, and body composition in Indian children: an in-silico study
Source: Front Nutr. 2025 Mar 27;12:1519756. doi: 10.3389/fnut.2025.1519756 (PMC11983558; doi:10.3389/fnut.2025.1519756)
Supplement: Supplementary file 1 [file Table_1.DOCX]

**SUPPLEMENTARY MATERIAL**

**Additional details of ONS-1 and ONS-2 composition:**

| Parameter | Details |
| --- | --- |
| Glycaemic Index (GI) | Low GI of 55(1) |
| Manufacturing Process | Utilizes natural cereal grain enzymes to produce complex carbohydrates with prebiotic benefits |
| Daily Serving Size | 54 g of powder in 300 ml milk (27 g in 150 ml milk twice daily) |
| Energy Content (per serve in milk) | 422 Kcal (~18% of EAR*) |
| Macronutrient Composition (aligned with AMDR guidelines) |  |
| - Protein | 15.7 g (~46% of RDA), contributing 15% of energy |
| - Fat | 13 g (~35% of RDA), contributing 28% of energy |
| - Carbohydrates | 57.5 g (~44% of RDA), contributing 54% of energy |
| Fat Content (as powder) | 1.1 g of fat/day (54 g) |
| Saturated Fat (per daily serve) | 0.9 g |
| Trans-fat (per daily serve) | Negligible (0.04 g) |
| Cholesterol (per daily serve) | < 5 mg |
| Sodium (per daily serve) | 243 mg (15% of RDA) |
| Micronutrient Composition | Delivers 26 vitamins and minerals |

*Estimated average requirement.

**Supplementary tables:**

Table S1: Baseline Anthropometric and Body Composition Parameters Across All Groups.

| **Outcome Parameters** | **Baseline** |
| --- | --- |
| Growth percentiles | 3^rd^ to 15^th^ |
| Height (cm) | 125.2 ± 14.7 |
| Weight (kg) | 23.5 ± 7.8 |
| BMI (kg/m^2^) | 14.5 ± 1.2 |
| Fat free mass (kg) | 20.5 ± 6.4 |
| Fat mass (kg) | 3 ± 1.9 |
| Bone mineral content (g) | 797 ± 229 |

Table S2: Anthropometric and Body Composition Values (Mean ± SD) at 12 Months:

| **Parameters** | **Group 1** | **Group 2** | **Group 3** | **Group 4** | **Group 5** | **Group 6** | **Group 7** |
| --- | --- | --- | --- | --- | --- | --- | --- |
| Height (cm) | 125.9 ± 14.9 | 127.4 ± 14.7 | 128.4 ± 14.6 | 127.2 ± 14.2 | 130.5 ± 14 | 129.3 ± 13.7 | 130.3 ± 14 |
| Weight (kg) | 23.4 ± 7.8 | 25.1 ± 8.2 | 25.9 ± 8.3 | 24.9 ± 7.7 | 27.5 ± 8.2 | 26.5 ± 7.8 | 27.5 ± 8.2 |
| BMI (kg/m2) | 14.5 ± 1.2 | 15 ± 1.2 | 15.2 ± 1.2 | 14.9 ± 1.1 | 15.7 ± 1.2 | 15.4 ± 1.1 | 15.7 ± 1.2 |
| Fat free mass (kg) | 20.4 ± 6.4 | 21.7 ± 6.7 | 22.4 ± 6.9 | 21.5 ± 6.4 | 23.7 ± 7 | 22.7 ± 6.6 | 23.6 ± 7 |
| Fat mass (kg) | 3 ± 2 | 3.4 ± 2.2 | 3.5 ± 2.1 | 3.4 ± 1.9 | 3.8 ± 2.1 | 3.8 ± 2 | 3.8 ± 2.1 |
| Bone mineral content (g) | 598 ± 185 | 886 ± 282 | 1007 ± 328 | 852 ± 220 | 1074 ± 322 | 906 ± 220 | 1072 ± 321 |

Table S3: Statistical comparison of between-group biochemical outcomes at different study timepoints.

| **Parameters** | **4 months** | | | **8 months** | | | **12 months** | | |
| --- | --- | --- | --- | --- | --- | --- | --- | --- | --- |
|  | **Group 5 vs Group 4** | | | | | | | | |
|  | **t-stats** | **p-value** | **Cohen's D** | **t-stats** | **p-value** | **Cohen's D** | **t-stats** | **p-value** | **Cohen's D** |
| Serum Calcium | 6 | < 0.0001 | 0.2 | 11 | < 0.0001 | 0.4 | 16 | < 0.0001 | 0.5 |
| Serum Ferritin | 53 | < 0.0001 | 1.7* | 95 | < 0.0001 | 3* | 117 | < 0.0001 | 3.7* |
| Urinary Iodine | 18 | < 0.0001 | 0.6* | 32 | < 0.0001 | 1* | 47 | < 0.0001 | 1.5* |
| Serum Selenium | 72 | < 0.0001 | 2.3* | 106 | < 0.0001 | 3.3* | 138 | < 0.0001 | 4.4* |
| Serum Vitamin A | 104 | < 0.0001 | 3.3* | 137 | < 0.0001 | 4.3* | 189 | < 0.0001 | 6* |
| Serum Vitamin B9 | 23 | < 0.0001 | 0.7* | 40 | < 0.0001 | 1.3* | 55 | < 0.0001 | 1.8* |
| Serum Vitamin B12 | 72 | < 0.0001 | 2.3* | 190 | < 0.0001 | 6* | 285 | < 0.0001 | 9* |
| Serum Vitamin D | 58 | < 0.0001 | 1.8* | 118 | < 0.0001 | 3.7* | 172 | < 0.0001 | 5.4* |
|  | **Group 5 vs Group 7** | | | | | | | | |
| Serum Calcium | 0 | 0.699 | 0 | 0 | 0.88 | 0 | 0 | 0.95 | 0 |
| Serum Ferritin | 0 | 0.682 | 0 | 1 | 0.574 | 0 | 1 | 0.427 | 0 |
| Urinary Iodine | 0 | 0.634 | 0 | 1 | 0.4 | 0 | 1 | 0.297 | 0 |
| Serum Selenium | 7 | < 0.0001 | 0.2 | 15 | < 0.0001 | 0.5 | 22 | < 0.0001 | 0.7* |
| Serum Vitamin A | 26 | < 0.0001 | 0.8* | 40 | < 0.0001 | 1.3* | 59 | < 0.0001 | 1.9* |
| Serum Vitamin B9 | 9 | < 0.0001 | 0.3 | 19 | < 0.0001 | 0.6* | 35 | < 0.0001 | 1.1* |
| Serum Vitamin B12 | 51 | < 0.0001 | 1.6* | 103 | < 0.0001 | 3.3* | 155 | < 0.0001 | 4.9* |
| Serum Vitamin D | 38 | < 0.0001 | 1.2* | 75 | < 0.0001 | 2.4* | 113 | < 0.0001 | 3.6* |

**Modelling framework – Equations and Calculations:**

The following section outlines the step-wise process for the calculations used to derive the predicted output parameters. This explains how the model is defined, incorporating key variables, and linking energetics to correction factors to obtain the final predictions.

Terminologies:

BMR Basal metabolic rate in kcal/day

BMC Bone mineral content in kg

BF% Body fat %

BMI Body mass index in kg/m^2^

BW Body weight in kg

C Carbohydrate (% or g)

CI Carbohydrate intake rate in kcal/day

∂BW Change in body weight in kg

∂FM Change in fat mass in (kg or kcal)

∂FFM Change in fat-free mass in (kg or kcal)

EI Energy intake in kcal/day

ER Energy requirement in kcal/kg/day

F Fat (% or g)

FI Fat intake rate in kcal/day

FM Fat mass in kg

FM' Fat mass rate in (kg/day or kcal/day)

FFM Fat-free mass in kg

FFM' Fat-free mass rate in (kg/day or kcal/day)

M/F Male/Female

P Protein (% or g)

p Partition co-efficient

PAE Physical activity energy expenditure in kcal/day

PI Protein intake rate in kcal/day

PAL Physical activity level

TEE Total energy expenditure in kcal/day

TEF Thermic effect of food in kcal/day

1. System definition parameters for the model:

a) Age (years)

b) Gender

c) BF%

d) Height (cm)

e) BW

1. User inputs for the model:
2. Duration: 4, 8 and 12 months
3. Physical activity level: As defined by the Indian Council of Medical Research (ICMR).
4. Energy intake: Determined based on the recommended energy requirement per kilogram of body weight per day.
5. Intervention: No intervention provided in case of macronutrient benchmarking.
6. Energetics equations and integration of correction factors:

TEF, which is the energy associated with the digestion, absorption, and short-term storage of macronutrients is calculated, as formulated by K.D. Hall(2),

TEF = (0.025*FI) + (0.25*PI) + (0.075*CI)

BMR, which is the body's energy expenditure at rest, essential for basic physiological functions, was estimated as follows:

TEE = TEF + BMR + PAE

= TEF + BMR + BMR (PAL -1)

BMR = (TEE-TEF)/PAL

BMR values were evaluated for different age groups and across percentiles (3^rd^ to 97^th^) for both genders.

This formula below, reflects the total energy expended by the body, accounting for the energy used in digestion, the energy needed for basic bodily functions at rest, and the additional energy expenditure due to physical activity.

TEE = TEF*(BMR+PAL)

The equation for energy for growth can be expressed as:

Energy for growth/Imbalance = EI - TEE

EI = (Energy requirement * BW)*ƞ

The partition coefficient estimation involves the following steps:

1. Calculating the daily change in fat mass and fat-free mass:

FM' (kg/day) = (Final FM - Initial FM)/365

FFM' (kg/day) = (Final FFM - Initial FFM)/365

1. Converting the daily changes in mass to energy expenditure in kilocalories per day:

FM' (kcal/day) = FM' (kg/day) *9400

FFM' (kcal/day) = FFM' (kg/day) *((4.3*(Initial FFM +837)

1. Calculating the imbalance:

Imbalance = FM' (kcal/day) + FFM' (kcal/day) = EI - TEE

These equations are applied in their delta (change over time) form rather than differential (instantaneous rate of change) form, as per the approach by K.D. Hall(3).

1. Finally, determining the partition co-efficient:

p = (FFM' (kcal/day) / Imbalance) * Ɛ

The 'p' values were evaluated for different age groups and across percentiles (3^rd^ to 97^th^) for both genders. The steps below provide insights into how energy is partitioned between fat-free mass and fat mass changes during growth:

1. Change in FFM:

∂FFM (kcal) = p*(EI – TEE)

∂FFM (kg) = ∂FFM (kcal) / ((4.3* FFM (kg)) +837)

Adding ∂FFM (kg) to initial FFM to get new FFM:

New FFM (kg)= Initial FFM + ∂FFM (kg)

1. Change in FM:

∂FM (kcal) = (1-p) * (EI – TEE)

∂FM (kg) = ∂FM (kcal)/9400

Adding ∂FM (kg) to initial FM to get new FM:

New FM (kg)= Initial FM + ∂FM (kg)

1. Change in BW:

∂BW (kg) = ∂FM (kg) + ∂FFM (kg)

Adding ∂BW (kg) to initial BW to get new BW:

New BW (kg)= Initial BW + ∂BW (kg)

Similarly, new BF% can be calculated as,

New BF% = New FM (kg)*100/New BW (kg)

Height is calculated as,

Height and weight correlation = (1/k) *ln (BW/A)

where,

A and k: co-efficient of exponential equation

New height = Initial height + delta Height and weight correlation

BMI is calculated using the formula:

BMI = BW/(Height/100)^2^

BMC is determined based on the individual's BMI using the following criteria:

BMC = [(3.7*BW)/100]*β

**Supplementary figures:**

Micronutrients linked to growth outcomes

Figure S1 illustrates the associations of macronutrients (protein) and micronutrients (calcium, zinc, iron, and vitamins B9, B12, and D) with key indicators of children's body composition and growth, such as fat mass, bone mass, muscle mass, and linear growth. The figure emphasizes the impacts of adequate intake as well as the nutrient deficiencies reported in the CNNS 2018 survey, on anthropometric and body composition parameters.

Linear growth (height for age) is linked to adequate intake of protein and essential micronutrients, including calcium, zinc, iron, and vitamins B9, B12, and D. Protein intake is shown to correlate positively with muscle mass, influencing overall body water content (represented by the dashed area), which is an indirect marker of muscle development.

Similarly, weight for age was influenced by changes in body fat mass and fat-free mass (muscle and bone mass). Protein, along with calcium, iron, and vitamins B9 and B12 was positively linked to fat mass. Bone mass was similarly enhanced by protein, calcium, and vitamin D. Muscle mass was particularly responsive to protein, calcium, zinc, iron, and vitamin D.


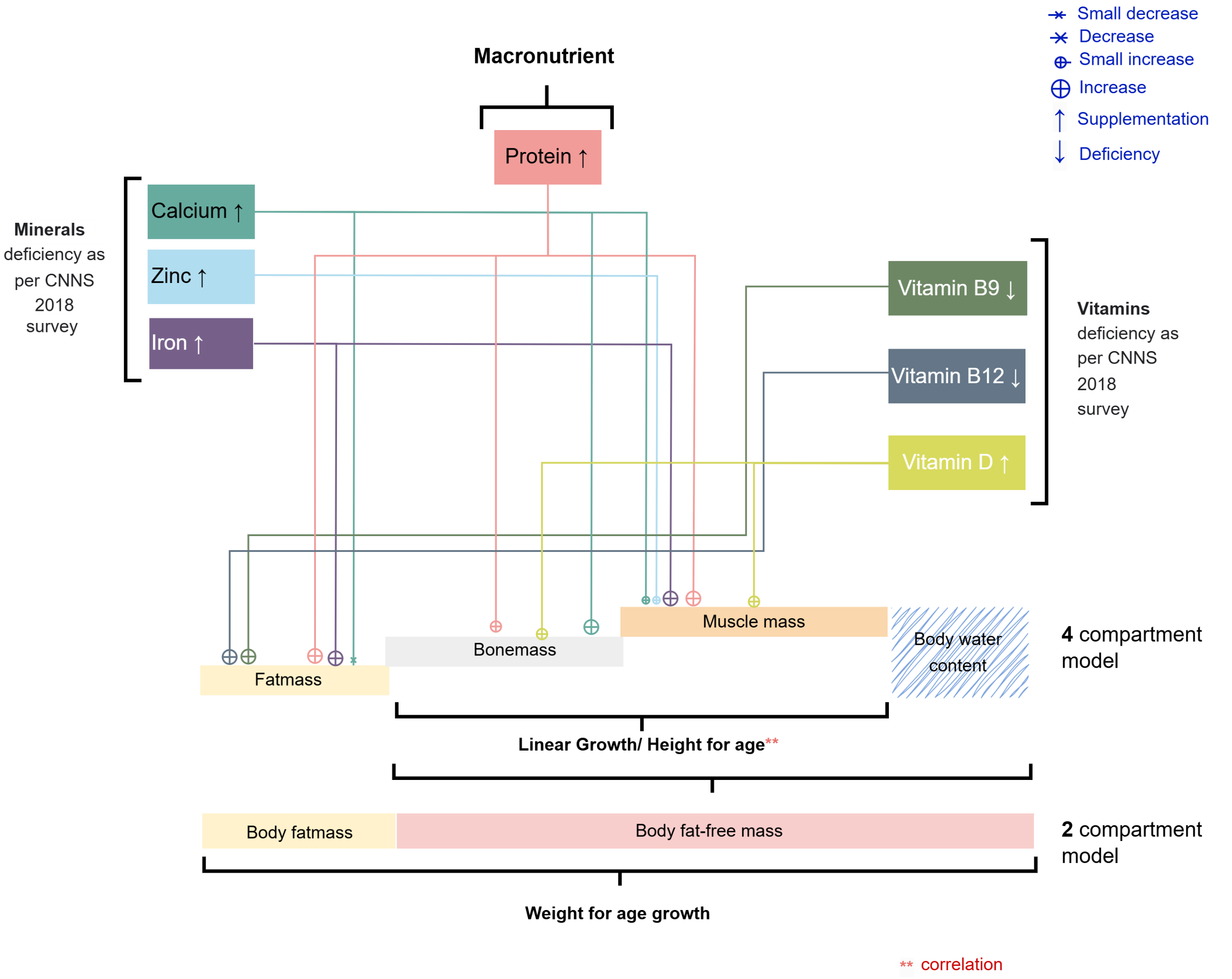


Figure S1. Association of micronutrients with physical growth outcomes based on published studies.

Model benchmarking against IAP 2015 growth trajectories

Figure S2 shows the illustrative trends of 3rd, 10th and 50th percentiles for various ages for both boys (Figure S2a) and girls (Figure S2b). The model with the set parameter could trace the growth trajectory for various percentiles with <10% and <3% error for height and weight respectively.





Figure S2. Prediction of height and weight growth trajectories by gender and growth percentiles, benchmarked against IAP 2015 data for children aged 6 to 17 years. a) Boys; b) Girls. Solid lines: Height, Dotted lines: Weight. Red: 3^rd^ percentile; Orange: 10^th^ percentile; Green: 50^th^ percentile.

Model simulation case studies

The model could predict a specific intervention effect over a specific dosage and duration. Figure S3 Case 1a and S3 Case 1b shows the effect of 100 kcal intervention above the basal diet for a 7-year boy in the 10th percentile of weight. The model predicts that the intervention may result in 4cm growth in height over a year and 2.4 kg growth in weight (Figure S3 Case1b). The growth was only due to the macronutrient (66% carbohydrate, 24% fat and 8% protein) as the effect of micronutrients was not accounted for in the model prediction. This results in a height and weight velocities of 0.34 cm/month and 202 g/month, which is a reasonable growth as obtained from the IAP data.

Similarly, protein supplementation (Figure S3 Case 2a and S3 Case 2b) accounting for 12% of total calories was administered to girls aged 12 years in the 10th percentile for height along with 100% RDA intake of micronutrients, would result in a height growth of 2.5 cm and a weight gain of 2.5 kg over a year. This translates to a height velocity of 0.21 cm/month and a weight velocity of 200 g/month, respectively, showing a percent shift of 1.79% in height and 8.1% in weight. Thus, the model in principle can be used to monitor the growth trajectory of children and predict the possible growth outcomes due to an intervention of macro and micronutrient supplementation.


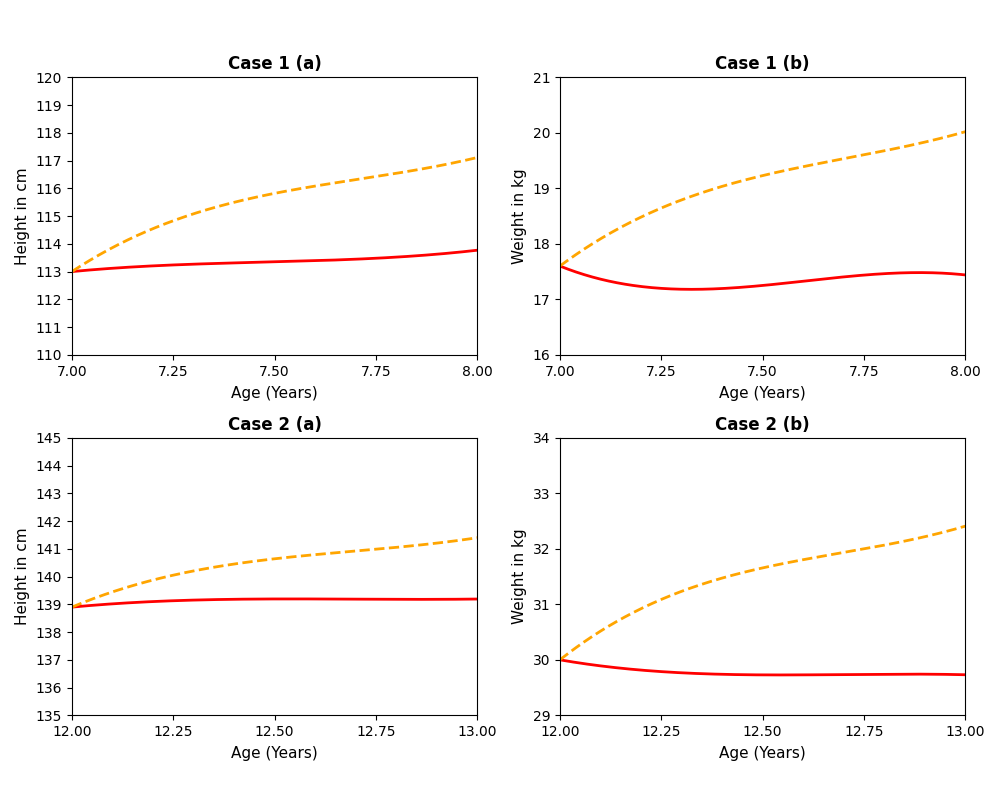


Figure S3. Model simulation case studies. Case 1 depicts a 7-year-old boy of the 10^th^ percentile receiving a 100 kcal/day intervention in addition to his basal diet. Case 2 represents a 12-year-old girl of the 10^th^ percentile receiving protein supplementation constituting 12% of total calories, along with micronutrient supplementation meeting 100% of the RDA. (a) Height; (b) Weight. Red solid line: Growth trajectory without intervention. Yellow dotted line: Growth trajectory with intervention.

Validation of the biochemical profile modelling platform

The biochemical platform was validated using published nutrient intervention trials in children. Figure S4 illustrates the validation of the biochemical platform by comparing the predicted and observed fold changes in serum nutrient levels, using data from published clinical trials. The tight clustering of points around the line suggests that the model provides reliable predictions for biochemical changes under different intervention conditions. The published intervention trials assessing serum levels of zinc(4–8), ferritin(9–13), vitamin D(14–17), calcium(18,19), and B-vitamins(20,21), were very well predicted by the model. The error percentages were as follows: zinc: 6.74%, ferritin: 7.9%, B-complex: 13%, calcium: 4.9%, and vitamin D: 5.9%


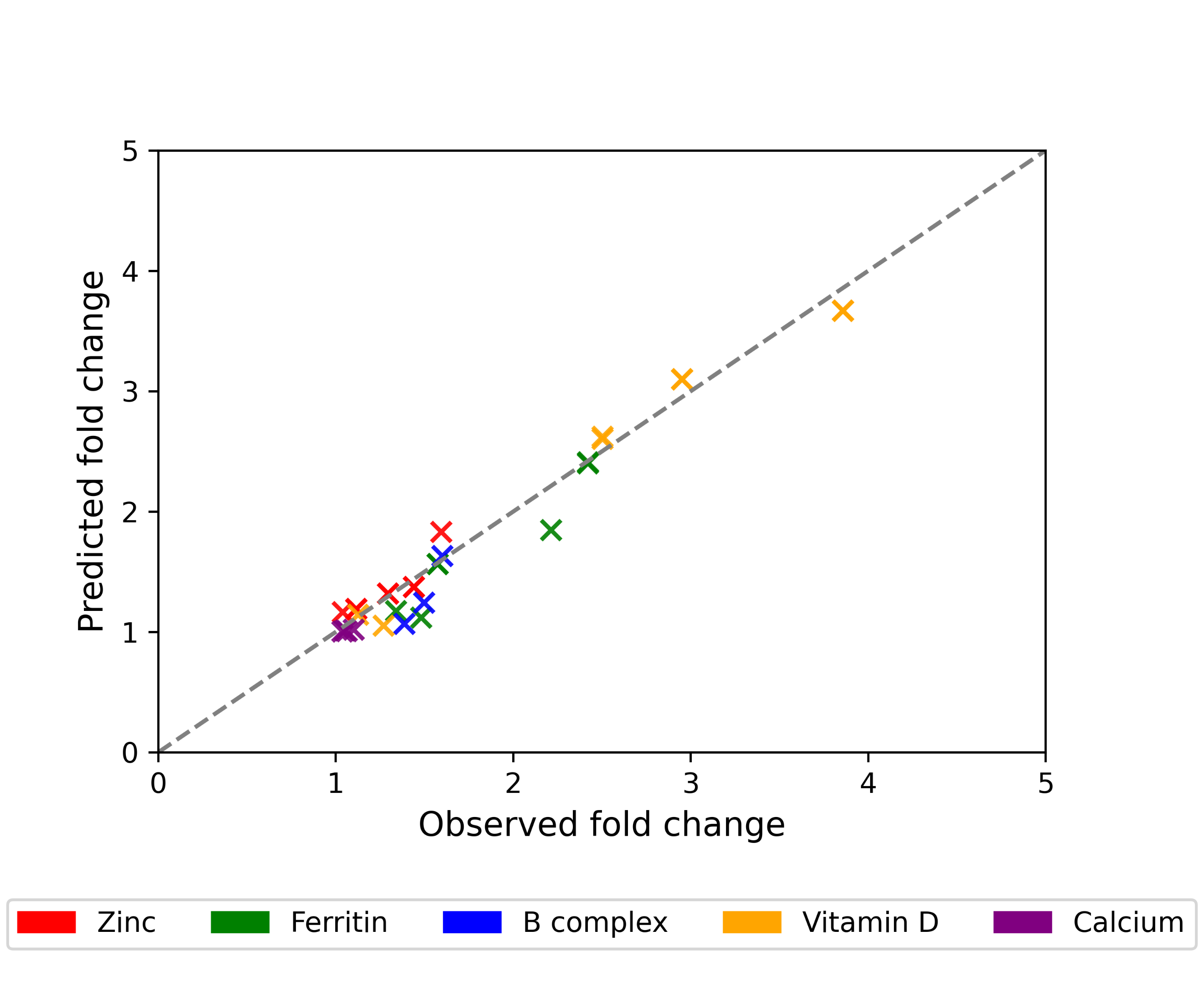


Figure S4. Validation of the biochemical platform using published clinical trials.

Impact of Horlicks intervention on serum nutrient levels


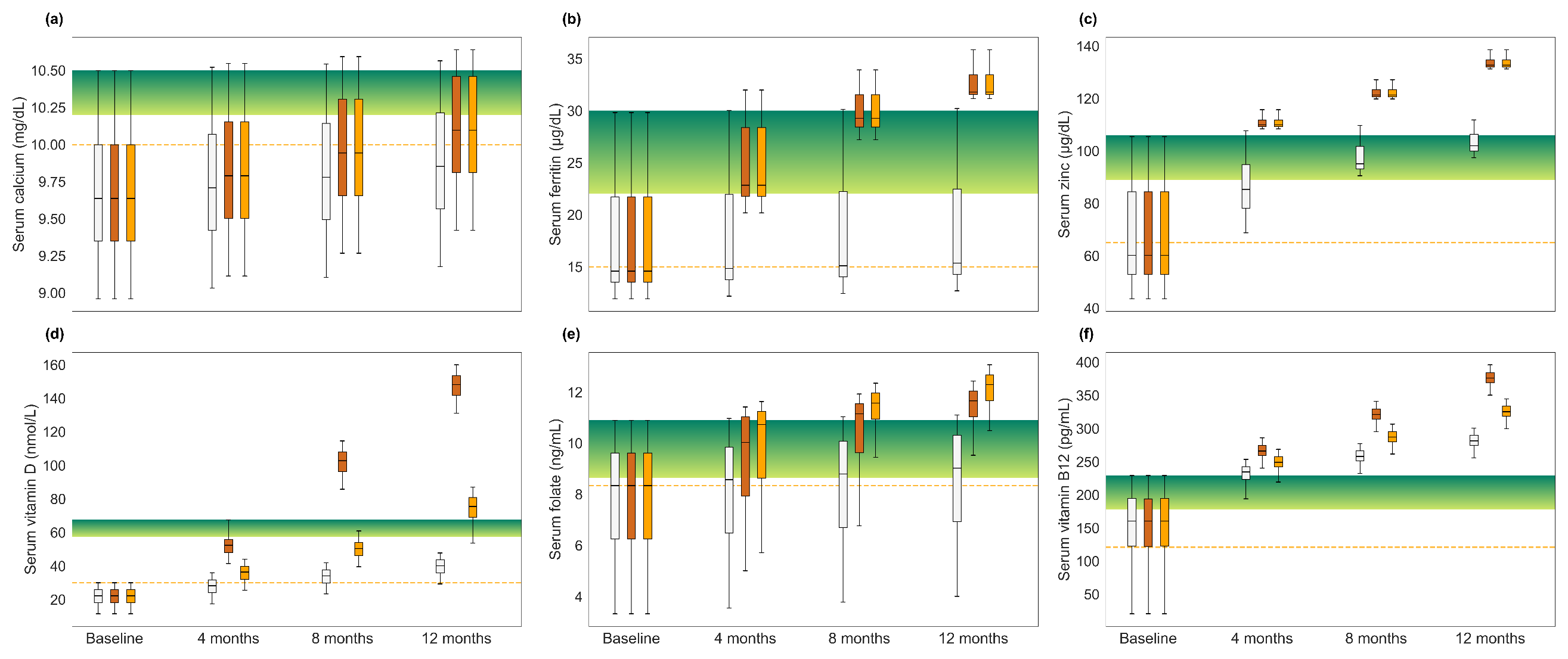


Figure S5. Post-intervention changes in biochemical parameters (w.r.t. baseline) among groups 4, 5 and 7. (a) Serum calcium levels; (b) Serum ferritin levels; (c) Serum zinc levels; (d) serum vitamin D levels; (e) serum vitamin B9 levels; (f) serum vitamin B12 levels. Box: interquartile range; top, middle, and bottom edge of the box: Q1, Q2, Q3 respectively. White: Group 4; Brown: Group 5; Orange: Group 7.

Figure S5 compares post-intervention biochemical profiles between the groups 4 (milk only group), 5 (new Horlicks group) and 7 (market-available Horlicks). The dosage of vitamin B9, vitamin B12 and vitamin D was changed by -29%, +46%, and +112% respectively in the Horlicks new formulation compared to market-available Horlicks, which is reflected in the 12^th^ month serum levels of these nutrients. The deficiency cut-offs for key micronutrients are: serum calcium (10.7 mg/dL)(22), serum ferritin (15 µg/L)(23), serum zinc deficiency (65 µg/dL)(24), serum vitamin D deficiency (20 nmol/L)(22), serum vitamin B9 (6-20 ng/mL)(23), and serum vitamin B12 (203 pg./mL)(24).

Comparison of post-intervention effects between Group 5 and Group 7


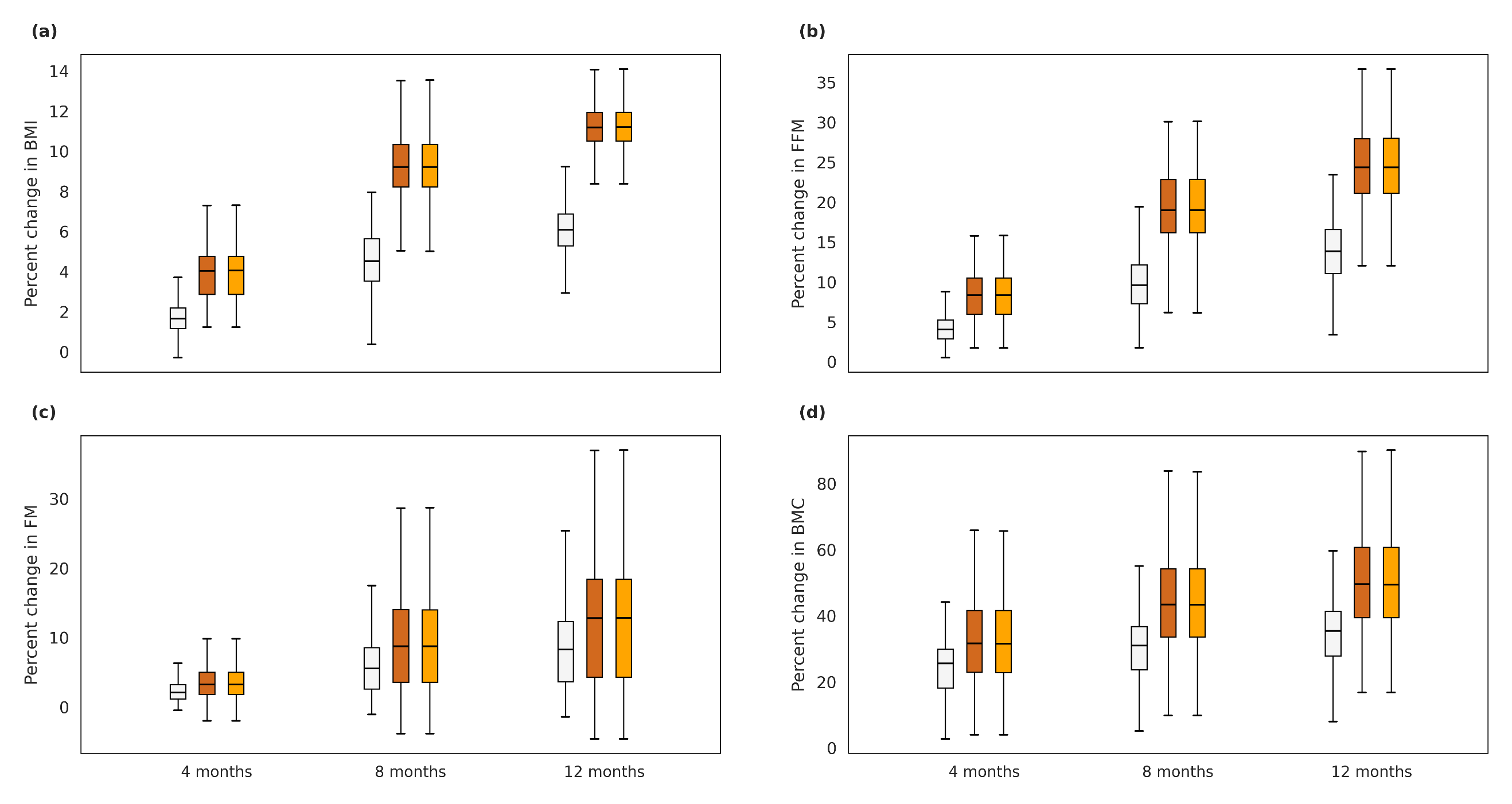


Figure S6. Post-intervention changes in BMI and body composition parameters (w.r.t. baseline) among group 5 and group 7. (a) Percent change in BMI. (b) Percent change in fat-free mass. (c) Percent change in fat mass. (d) Percent change in BMC (bone mineral content). Box: interquartile range; top, middle, and bottom edge of the box: Q1, Q2, Q3 respectively Brown: Group 5 Group; Orange: Group 7.

The *in-silico* cohort simulated the effects of new Horlicks and market-available Horlicks by comparing group 5 and group 7 respectively. Comparison of group 5 and group 7 yielded a Cohen’s D value of 0 indicating no significant difference between the two groups in terms of growth outcomes. Both the groups led to average increases of 265 g of FFM, 72 g of FM and 13 g of BMC by the 12^th^ month of intervention. Although Vitamin D, and vitamin B12 were increased in dosages while vitamin B9 dosage was lower in group 5 compared to group 7 (Table 2), both compositions satisfied more than 75% RDA, leading to similar outcomes for both groups.

**References:**

1. Khanna D, Bhatt J, Gupta J, Sethi S, Joshi P, Pareek M, Agrawal D. Glycemic Indices of Multiple Oral Nutritional Supplements: A Randomized Cross-Over Study in Indian Adults. Food and Nutrition Sciences. 2023 Oct 11;14(10):941-62. https://doi.org/10.4236/fns.2023.1410060

2. Hall KD. Predicting metabolic adaptation, body weight change, and energy intake in humans. American Journal of Physiology-Endocrinology and Metabolism. 2010 Mar;298(3):E449-66. https://doi.org/10.1152/ajpendo.00559.2009

3. Hall KD, Butte NF, Swinburn BA, Chow CC. Dynamics of childhood growth and obesity: development and validation of a quantitative mathematical model. The lancet Diabetes & endocrinology. 2013 Oct 1;1(2):97-105. https://doi.org/10.1016/S2213-8587(13)70051-2

4. Chiplonkar SA, Kawade R. Effect of zinc-and micronutrient-rich food supplements on zinc and vitamin A status of adolescent girls. Nutrition. 2012 May 1;28(5):551-8. https://doi.org/10.1016/j.nut.2011.08.019

5. Gibson RS, Vanderkooy PD, MacDonald AC, Goldman A, Ryan B, Berry M. A growth-limiting, mild zinc-deficiency syndrome in some southern Ontario boys with low height percentiles. The American journal of clinical nutrition. 1989 Jun 1;49(6):1266-73. https://doi.org/10.1093/ajcn/49.6.1266

6. Udomkesmalee E, Dhanamitta S, Sirisinha S, Charoenkiatkul S, Tuntipopipat S, Banjong O, Rojroongwasinkul N, Kramer TR, Smith Jr JC. Effect of vitamin A and zinc supplementation on the nutriture of children in Northeast Thailand. The American journal of clinical nutrition. 1992 Jul 1;56(1):50-7. https://doi.org/10.1093/ajcn/56.1.50

7. Guevara DA, Reyes S, López M, Flores N, Aguirre S, Muñoz EB, Fornasini M, Baldeón ME. Impact of milk based micronutrient supplementation in school children in Quito-Ecuador. Nutricion hospitalaria. 2018;35(1):50-8. https://doi.org/10.20960/nh.1353

8. Chen K, Zhang X, Li TY, Chen L, Wei XP, Qu P, Liu YX. Effect of vitamin A, vitamin A plus iron and multiple micronutrient-fortified seasoning powder on infectious morbidity of preschool children. Nutrition. 2011 Apr 1;27(4):428-34. https://doi.org/10.1016/j.nut.2010.04.004

9. Rosado JL, Lopez P, Muñoz E, Martinez H, Allen LH. Zinc supplementation reduced morbidity, but neither zinc nor iron supplementation affected growth or body composition of Mexican preschoolers. The American journal of clinical nutrition. 1997 Jan 1;65(1):13-9. https://doi.org/10.1093/ajcn/65.1.13

10. Vaz M, Pauline M, Unni US, Parikh P, Thomas T, Bharathi AV, Avadhany S, Muthayya S, Mehra R, Kurpad AV. Micronutrient supplementation improves physical performance measures in Asian Indian school-age children. The Journal of nutrition. 2011 Nov 1;141(11):2017-23. https://doi.org/10.3945/jn.110.135012

11. Andersson, M., Thankachan, P., Muthayya, S., Goud, R. B., Kurpad, A. V., Hurrell, R. F., & Zimmermann, M. B. (2008). Dual fortification of salt with iodine and iron: a randomized, double-blind, controlled trial of micronized ferric pyrophosphate and encapsulated ferrous fumarate in southern India. *The American journal of clinical nutrition*, *88*(5), 1378-1387. https://doi.org/10.3945/ajcn.2008.26149

12. Siddiqui IA, Rahman MA, Jaleel A. Efficacy of daily vs. weekly supplementation of iron in schoolchildren with low iron status. Journal of tropical pediatrics. 2004 Oct 1;50(5):276-8. https://doi.org/10.1093/tropej/50.5.276

13. Angeles IT, Schultink WJ, Matulessi P, Gross R, Sastroamidjojo S. Decreased rate of stunting among anemic Indonesian preschool children through iron supplementation. The American journal of clinical nutrition. 1993 Sep 1;58(3):339-42. https://doi.org/10.1093/ajcn/58.3.339

14. Economos CD, Moore CE, Hyatt RR, Kuder J, Chen T, Meydani SN, Meydani M, Klein E, Biancuzzo RM, Holick MF. Multinutrient-fortified juices improve vitamin D and vitamin E status in children: a randomized controlled trial. Journal of the Academy of Nutrition and Dietetics. 2014 May 1;114(5):709-17. https://doi.org/10.1016/j.jand.2013.07.027

15. El-Hajj Fuleihan G, Nabulsi M, Tamim H, Maalouf J, Salamoun M, Khalife H, Choucair M, Arabi A, Vieth R. Effect of vitamin D replacement on musculoskeletal parameters in school children: a randomized controlled trial. The Journal of Clinical Endocrinology & Metabolism. 2006 Feb 1;91(2):405-12. https://doi.org/10.1210/jc.2005-1436

16. Stounbjerg NG, Thams L, Hansen M, Larnkjær A, Clerico JW, Cashman KD, Mølgaard C, Damsgaard CT. Effects of vitamin D and high dairy protein intake on bone mineralization and linear growth in 6-to 8-year-old children: the D-pro randomized trial. The American journal of clinical nutrition. 2021 Dec 1;114(6):1971-85. https://doi.org/10.1093/ajcn/nqab286

17. Marwaha RK, Garg MK, Sethuraman G, Gupta N, Mithal A, Dang N, Kalaivani M, Ganie MA, Narang A, Arora P, Singh A. Impact of three different daily doses of vitamin D3 supplementation in healthy schoolchildren and adolescents from North India: a single-blind prospective randomised clinical trial. British Journal of Nutrition. 2019 Mar;121(5):538-48. https://doi.org/10.1017/s0007114518003690

18. Khadilkar AV, Sayyad MG, Sanwalka NJ, Bhandari DR, Naik S, Khadilkar VV, Mughal M. Vitamin D supplementation and bone mass accrual in underprivileged adolescent Indian girls. Asia Pacific journal of clinical nutrition. 2010 Dec;19(4):465-72.

19. Suryono S, Setiawan B. The effects of milk consumption on blood calcium concentration and bone density of adolescents boys. Indonesian Food Science and Technology Journal. 2021 Dec 31;5(1):12-6.

20. Kumar MV, Rajagopalan S. Impact of a multiple-micronutrient food supplement on the nutritional status of schoolchildren. Food and nutrition bulletin. 2006 Sep;27(3):203-10. https://doi.org/10.1177/156482650602700302

21. Dehkordi EH, Sedehi M, Shahraki ZG, Najafi R. Effect of folic acid on homocysteine and insulin resistance of overweight and obese children and adolescents. Advanced biomedical research. 2016 Jan 1;5(1):88. https://doi.org/10.4103/2277-9175.182219

22. Gupta P, Dabas A, Seth A, Lakshmi Bhatia V, Khadgawat R, Kumar P, et al. Recommendations Indian Academy of Pediatrics Revised (2021) Guidelines on Prevention and Treatment of Vitamin D Deficiency and Rickets.

23. Chandra J, Dewan P, Kumar P, Mahajan A, Singh P, Dhingra B, Radhakrishnan N, Sharma R, Manglani M, Rawat AK, Gupta P. Diagnosis, treatment and prevention of nutritional anemia in children: recommendations of the joint committee of pediatric hematology-oncology chapter and pediatric and adolescent nutrition society of the Indian Academy of Pediatrics. Indian pediatrics. 2022 Oct;59(10):782-801.

24. Ministry of Health and Family Welfare (MoHFW), Government of India, UNICEF and Population Council. 2019. Comprehensive National Nutrition Survey (CNNS) National Report. New Delhi. 2019
